# Identifying the active sites in unequal iron-nitrogen single-atom catalysts

**DOI:** 10.1038/s41467-023-41311-9

**Published:** 2023-09-11

**Authors:** Liang Huang, Qiong Liu, Weiwei Wu, Ge Gao, Xiliang Zheng, Jin Wang, Shaojun Dong

**Affiliations:** 1 State Key Laboratory of Electroanalytical Chemistry, Changchun Institute of Applied Chemistry, Chinese Academy of Sciences, Changchun, 130022 China; 2 School of Applied Chemistry and Engineering, University of Science and Technology of China, Hefei, 230026 China; 3 Center for Theoretical Interdisciplinary Sciences Wenzhou Institute, University of Chinese Academy of Sciences, Wenzhou, 325001 China; 4 Department of Chemistry and Physics, State University of New York at Stony Brook, Stony Brook, NY 11794-3400 USA

**Keywords:** Electrocatalysis, Heterogeneous catalysis, Electrocatalysis

## Abstract

Single-atom catalysts (SACs) have become one of the most attractive frontier research fields in catalysis and energy conversion. However, due to the atomic heterogeneity of SACs and limitations of ensemble-averaged measurements, the essential active sites responsible for governing specific catalytic properties and mechanisms remain largely concealed. In this study, we develop a quantitative method of single-atom catalysis–fluorescence correlation spectroscopy (SAC-FCS), leveraging the atomic structure-dependent catalysis kinetics and single-turnover resolution of single-molecule fluorescence microscopy. This method enables us to investigate the oxidase-like single-molecule catalysis on unidentical iron-nitrogen (Fe-N) coordinated SACs, quantifying the active sites and their kinetic parameters. The findings reveal the significant differences of single sites from the average behaviors and corroborate the oxidase-like catalytic mechanism of the Fe-N active sites. We anticipate that the method will give essential insights into the rational design and application of SACs.

## Abstract (web summary)

Intrinsic active sites of single-atom catalysts are often obscured in ensemble-averaged measurements. Here, the authors successfully quantified Fe-N active sites by employing single-molecule fluorescence microscopy combined with the atomic structure-dependent oxidase-like catalysis model.

## Introduction

Single-atom catalysts (SACs), with atomically dispersed metal centers anchored to the support matrices, have recently emerged as the most innovative heterogeneous catalysts in thermo-, photo- and electrocatalysis^1–7^. Compared to the nanomaterials, their maximized atom utilization efficiency and the simplified configurations of the catalytic sites show tremendous potential for the mechanism investigation and single-atom catalysis^8–11^. However, the heterogeneous substrates and coordination interactions tend to construct unidentical atomic structures in SACs, leading to multiple catalytic properties and reaction kinetics^12,13^. In particular, the widely studied iron-nitrogen coordinated (FeN_x_/C) SACs through uncontrollable pyrolysis will produce multiple active moieties with varying catalytic activities^14–17^. For instance, our previous research indicates the strong coordination structure-dependent catalytic activity^18^. The axial N-coordinated FeN_5_ active sites behaved like the heme and exhibited much higher oxidase-like activity than that of the coplanar FeN_4_ sites due to the electron push effects for oxygen molecule^19^. But restricted to the non-uniform atomic sites in SACs and the insufficient resolution of characterizations, the significant catalytic and kinetic properties of the active species are deeply blurred from the blended single-atom sites^8,12,20^, and the SACs technology encounters formidable challenges. Innovative techniques and methods are urgently needed to identify the actual active sites and the underlying mechanisms of single-atom catalysis.

For the heterogeneous catalysis on SACs, the molecular reaction kinetics are closely related to the atomic structures of different catalytic sites^10,13,19^, thus enabling the deconvolution of active sites from the kinetic behaviors of the single-molecule reaction^16,21^. Fortunately, fluorescence correlation spectroscopy (FCS), based on single-molecule fluorescence microscopy, can quantitatively evaluate the time-correlated fluorescence fluctuations at single-turnover resolution^22–25^. When taking fluorescent molecules as reactants/products, the fluorescence turnover trajectory, reflecting the catalytic kinetics of each individual site, facilitates the identification of multiple catalytic sites^26–28^.

Here, we develop a single-atom catalysis–fluorescence correlation spectroscopy (SAC-FCS) method and successfully resolve the real-time fluorescent trajectories of single-molecule oxidase-like catalysis on FeN_x_/C SACs by correlating the autocorrelation of single turnover reaction with unidentical atomic sites. Consequently, we can identify and quantify the intrinsic oxidase-like active centers from hybrid single-atom sites through the autocorrelation calculation of the SAC-FCS method. We uncover the significant atom and catalytic kinetic parameters of the FeN_5_ active site in FeN_x_/C SACs. The active site proportion of the oxidase-like active sites is about 26%, and the catalytic rate constant of the active site toward heterogeneous oxidase-like catalytic reaction can be 182 and 1463 times higher than that of the inactive sites in FeN_x_/C SACs and the FeN_4_ site in iron phthalocyanine, respectively.

## Results

### SAC-FCS method and mechanism

**Fig. 1:**
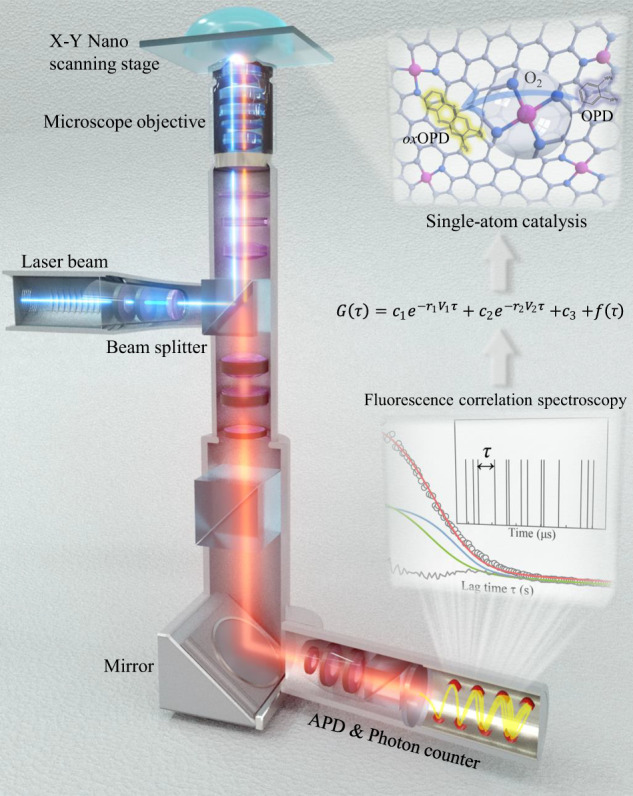
SAC-FCS setup and mechanism. Schematic of the single-atom catalysis model and setup of the fluorescence correlation spectroscopy, including the oxidase-like redox catalysis of single-atom sites, collection of single-molecule fluorescence signals, autocorrelation fitting and the identification of the atomic active sites. The measurements were carried out on a cover slide with a 5 × 5 mm cylindrical reaction cell at room temperature. FeN_x_/C SACs were anchored to the bottom of the reactor, and then the air-saturated 100 mM sodium acetate buffer with 1 nM o-phenylenediamine (OPD) was added to the cell. FeN_x_/C SACs catalyzed oxidation of the non-fluorescent OPD substrates into fluorescent products of 2,3-diaminophenazine (DAP). The generated photons from DAP were collected by photon counter (details are in Supplementary Information 2.1–2.3).

Different from the classical ensemble studies for the activity and kinetic parameters, where numerous underlying catalytic details and differences on atomic sites are hidden in ensemble-averaged measurements. The developed SAC-FCS method has combined the single-molecule fluorescence measurement with the autocorrelation spectrum calculation and can be used to quantitatively study the molecular reaction kinetics on a single site through real-time fluorescent observation of the individual chemical reactions^28–30^. As shown in Fig. 1, the nonfluorescent substrate is first converted into a fluorescent molecule via the heterogeneous catalysis on the single-atom site of the SACs, then the generated fluorescence signal in the illuminated region is detected by the avalanche photodiode (APD) and converted into electrical pulse signal^28^, which reflecting the information on the underlying photon dynamics^24,26^. Then, the arrival times of each photon are recorded by a photon counter^23^. Due to the extremely low substrate concentration, the time interval between two catalytic reactions in the measurement area is about the order of microseconds, far more than the time resolution of the FCS. Thus, all the catalytic reaction times in the measurement area can be recorded and distinguished. In consequence, the developed SAC-FCS method not only can monitor the entire atomic sites and catalytic process in the observation region but also simultaneously distinguishes each individual trajectory of fluorescence signal.

Specifically, the single-atom catalytic kinetics of the SACs are studied based on a heterogeneous redox reaction of an oxidase-like catalysis scheme. The non-fluorescent substrate of o-phenylenediamine (OPD) is oxidized into the fluorescent product of 2,3-diaminophenazine (DAP) with the catalysis of the SACs and O_2_ oxidant. Accordingly, the iron phthalocyanine (FePc) and FeN_x_/C are respectively set as the standard SACs model and practical SACs model due to their inherent high oxidase-like catalytic activity from the Fe-N_x_ coordinated single-atom sites^18^.

$${{{{{\rm{o}}}}}}-{{{{{\rm{phenylenediamine}}}}}}\,\left({{{{{\rm{OPD}}}}}}\right)+{O}_{2}\mathop{\to }\limits^{{{FeN}}_{x}}2,3 -{{{{{\rm{diaminophenazine}}}}}}\, \left({{{{{\rm{DAP}}}}}}\right)+{H}_{2}O$$

The catalytic behaviors of different FeN_x_ sites can be described by the single-turnover trajectories of DAP fluorescence signals from the SAC-FCS method. First, the arrival time of an individual photon represents the completion of single turnover catalysis on a single-atom site. Thus, the relevant kinetic processes are stochastic in the interval between any two arrival times, and the distribution of time interval is reflected from the autocorrelation spectrum of the catalytic sites^19,26^. Second, every single fluorescent molecule generated in the observation region is separately recorded by single-molecule fluorescence microscopy; namely, the corresponding catalytic kinetics of the different single-atom sites can be statistically reflected in the shape of the FCS curve. Then, we can successfully build the following theoretical SAC-FCS model.

According to the single-atom catalytic mechanism, the intrinsic catalytic property highly depends on the coordination structure and the central atom of the site. The autocorrelation process measures the time interval and lag time (*τ*) between the fluorescence events in single-molecule fluorescence signals (Supplementary Information 2.3). The higher activity of the catalytic site generates more distribution of the low lag time (*τ*) part^28,31^. Thus, the kinetic differences of the unequal single-atom sites can be distinguished from the distinct autocorrelation spectra. Generally, the catalytic kinetics of the active sites are several times to hundreds of times higher than that of the other inactive sites. To get more kinetic information of the active sites, the practical single-atom catalysis of SACs can be identified as the faster (*E*_*1*_) and slower (*E*_*2*_) process, based on the difference of their intrinsic redox catalytic activity. According to the simplified kinetic model of the single-atom oxidase-like reaction (the chemical equation and equilibrium model in Supplementary Information 2.3), the stochastic kinetics of single site *E*_1_ can be described as Eqs. 1–3 (as outlined in Supplementary Information 2.3).

1 $$\frac{d\left[E\right]}{d\left[t\right]}=-{k}_{1}\left[E\right]\left[S\right]+\left[{ES}\right]{k}_{-1}$$

2 $$\frac{d\left[{ES}\right]}{{dt}}={k}_{1}\left[E\right]\left[S\right]-\left[{ES}\right]\left({k}_{2}+{k}_{-1}\right)$$

3 $$\frac{d\left[P\right]}{{dt}}={k}_{2}\left[{ES}\right]$$

The concentrations of the active site ([*E*]), the intermediate complex ([*ES*]), fluorescent product ([*P*]), and non-fluorescent reactant molecule ([*S*]), as well as their correlations, are involved in the above equations, where *k*_1_ is the binding rate constant of the substrate on active site, *k*_-1_ is the dissociation rate of the intermediate complex, and *k*_2_ is the catalytic rate constant. The kinetics of this basic single-site model can be extended to the single molecule enzymology^31–34^. Thus, the autocorrelation equation corresponding to the kinetics of the single catalytic site (Supplementary Equations S4–S7) can be obtained through the derivation of these stochastic kinetic equations. Meanwhile, the kinetic parameters of a single-site catalytic process need to be modified with multi-site correlations because there is not only one FeN_x_ single-atom site in the observation region. The autocorrelation consists of a time-independent term and two exponential terms. The autocorrelation function can be a multi-exponential function or a stretched exponential function, which involves two separate kinetic processes, and the corresponding kinetic rate and proportion of each catalytic site lie in the exponential terms. The final form of the autocorrelation is written as the following equation (detailed derivation process in Supplementary Information 2.3 and Supplementary Equations S4–S20).

4 $$G\left(\tau \right)={c}_{1}{e}^{-{r}_{1}{V}_{1}\tau }+{c}_{2}{e}^{-{r}_{2}{V}_{2}\tau }+\frac{1}{N}{\left[1+\frac{\tau }{{\tau }_{D}}\right]}^{-1}{\left[1+\frac{\tau }{{\tau }_{D}}{\omega }^{2}\right]}^{-\frac{1}{2}}+{c}_{3}$$

In Eq. 4, the first two terms respectively represent the fast and slow reaction process catalyzed by different single-atom sites^28,35^. The third term accounts for the spatial diffusion effects. Through solving the above equations from the SAC-FCS experiment and autocorrelation fitting^28,36^, the relatively intrinsic parameters of the single-atom active sites (Supplementary Equations S21–S25), such as the active FeN_x_ ratio (*r*_1_), reaction rates (*v*_m_), Michaelis-Menten constants (*K*_m_) and other kinetics can be definitely worked out.

### SAC-FCS quantification of the model catalysts

**Fig. 2:**
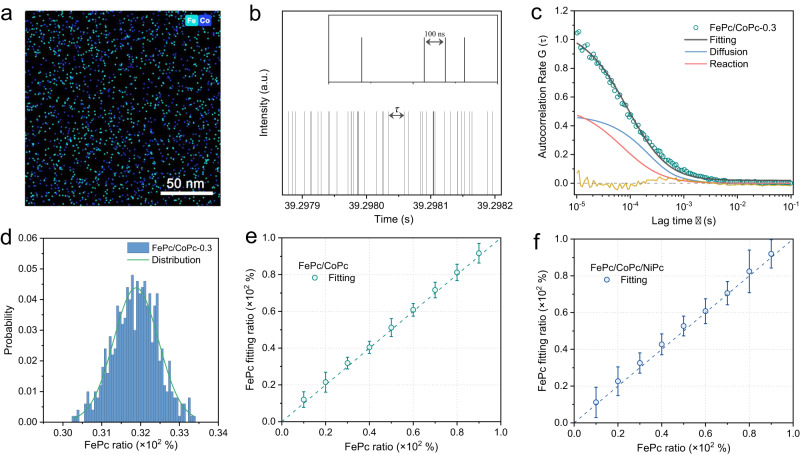
Single-atom models and SAC-FCS analysis. **a** Overlapped EDS mappings of the Fe and Co atomic distribution of the single-site metal phthalocyanine model. The cyan and blue pixels represent the Fe and Co element signals, respectively. **b** Single-molecule fluorescence signals, and **c** the experimental autocorrelation data and fitting curves of the FePc/CoPc-*0.3* model from the standard SAC-FCS method. The inset in (**b**) is the magnified fluorescence trajectory showing the high time resolution of the fluorescence correlation spectroscopy. **d** Histogram of the calculated FePc probability distribution from 500 times of the autocorrelation fitting. **e** Binary FePc/CoPc-*a* and **f** ternary FePc/CoPc/NiPc-*a* catalytic models with different SAC-FCS experimental and fitting ratios of the FePc active sites. The *a* ✕ 100% represents the actual percentage of FePc in the model, which ranges from 0.1 to 0.9.

We first established the standard catalytic model of single-site metal phthalocyanines to verify the SAC-FCS method (Fig. 2). The model is set up by uniformly immobilizing FePc with CoPc molecules (denoted as FePc/CoPc-*a*, where *a* = 0.1, 0.2…0.9, *a* ✕ 100% represents the percentage of FePc in the model) on the illuminated region, and carried out in microflow cells with the substrates of OPD and O_2_. The distribution of different catalytic centers within the scope can approximatively be seen as the two-dimensional SACs with unequal atomic sites (Fig. 2a and Supplementary Fig. 1). In addition, the minimum time resolution of the FCS is far below the microsecond time interval of the redox catalytic reaction on single-atom sites (Fig. 2b). The waiting time for the next arrival of fluorescent molecule is a stochastic variable and known as stationary residual time in renewal theory. In view of the multiple catalytic sites in the observation region, the probability density function for the different catalytic processes can be derived from differentiating the cumulative probability distribution for the waiting time. Thus, the fluorescence turnover trajectories reflect the unordered catalytic behaviors at a single-turnover resolution (Supplementary Fig. 2) and can be quantified through the statistical autocorrelation spectrum from the autocorrelation function. Finally, the experimental autocorrelation data fitting with Eq. 3 exhibits fine overlap and few residual errors (Fig. 2c and Supplementary Fig. 3), indicating that the autocorrelation equation effectively reveals the catalytic behaviors and kinetics of single-atom sites, and the contributions from free fluorescent diffusion (Supplementary Fig. 3b) and the oxidase-like reaction are also shown in the fitted curves.

As shown in Fig. 2d, after more than 500 times SAC-FCS analysis and fitting of the fluorescent trajectories from different illuminated regions, we obtain the statistical distribution of the active sites, namely the FePc, in standard sample of FePc/CoPc-*0.3*. The narrow distribution and convergent median are in close proximity to the actual content of 30% FePc. Meanwhile, by altering the FePc ratio in the FePc/CoPc system successively from 10% to 90% (Supplementary Fig. 4), the corresponding estimations quantified by the SAC-FCS method show excellent accuracies with small errors (Fig. 2e and Supplementary Fig. 5).

Furthermore, we evolve the single-site model of metal phthalocyanine to a more complicated ternary FePc/CoPc/NiPc to simulate the real SACs with multiple catalytic sites. Similarly, we conduct the single molecule fluorescence measurements to obtain the autocorrelation spectra (Supplementary Fig. 6). Due to the much higher oxidase-like activity than other phthalocyanine catalysts, the FePc acted as the intrinsic active sites in this catalytic reaction, which facilitates us to recognize and analyze the turnover trajectory of the real active centers. As expected, the proportion of FePc in this ternary system is well identified via the autocorrelation and fitting of the single fluorescence turnover trajectory, with different mixing ratios of the atomic sites (Fig. 2f and Supplementary Fig. 7). These indicate the tremendous potential of the SAC-FCS method for real-time probing the active center from multiple reaction kinetics. More importantly, it provides a significant approach for extended SACs research by deducing the atomic structure from single-molecule catalytic property.

### SAC-FCS application and extension

**Fig. 3:**
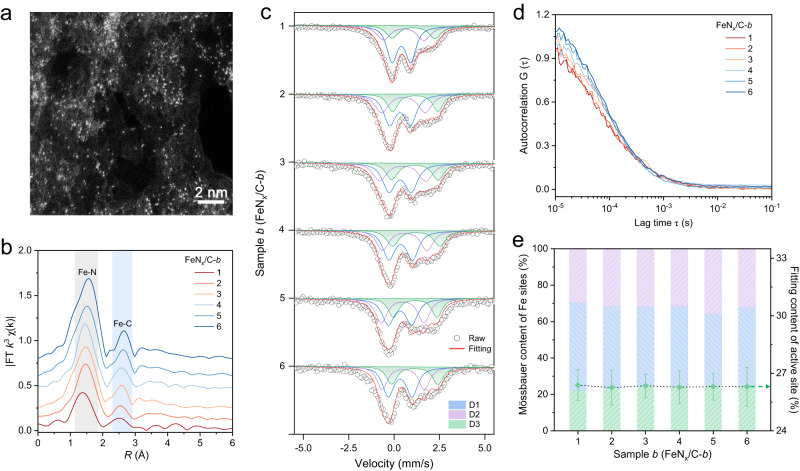
SAC-FCS identification of the active sites of FeN_x_/C SACs. **a** HAADF-STEM image of the Fe single atoms of FeN_x_/C-*3* SACs. **b** Fourier transformations for *k*^3^-weighted Fe K-edge EXAFS in R-space, **c** ^57^Fe Mössbauer spectra and **d** typical fluorescence autocorrelation of a series of FeN_x_/C-*b* SACs, *b* is defined as 1, 2…6. **e** The calculated ratio of FeN_5_ single-atom active sites in the corresponding FeN_x_/C-*b* SACs models from Mössbauer spectra and autocorrelation fitting, respectively.

We further extended the application of the SAC-FCS method to the FeN_x_/C SACs system (where x represents the first shell N-coordination number of Fe atom, x = 4, 5, 6), which consists of the atomically dispersed Fe coordinating with multiple N steric structures. According to the previous results, this would be one of the most typical examples for the actual SACs^18,37^. The FeN_x_/C SACs are delicately designed and synthesized through the successive metal-organic frameworks (MOFs) template growth, encapsulation and pyrolysis strategies (Supplementary Fig. 8). With the controllable synthesis, the FeN_x_/C SACs achieve the uniformities of the thickness, microstructure and atomic distribution along with the large-scale two-dimensional plane (Supplementary Fig. 9). As shown in the pore structure and composition characterization, the two-dimensional templates are reconstructed into Zn-MOFs with FePc molecules encapsulated in the larger cage units (Supplementary Fig. 10). Meanwhile, the aberration-corrected STEM (Fig. 3a) and EDS mappings exhibit the atomically dispersed Fe atoms over the carbon supports, with the Fe and the N atoms homogeneously distribute throughout the whole domain (Supplementary Fig. 11). The Fourier-transformed (FT) and wavelet transformed (WT) *k*^3^-weighted Fe K-edge extended X-ray absorption fine structure (EXAFS) spectra harvested from synchrotron X-ray absorption spectroscopy (XAS) indicate that the main peak of Fe K-edge at 1.50 Å is in accord with Fe-N scattering path (Supplementary Fig. 12), verifying the formation of atomically dispersed Fe-N_x_ centers with +2 ~ +3 valence Fe center in FeN_x_/C SACs^16,37^.

Through altering the amount of FePc precursor during the synthetic process, we obtained a series of FeN_x_/C-*b* SACs (*b* is defined as 1, 2…6, successively represents the increase of the atomic Fe loading) with different numbers of the Fe atomic sites, as well as the possible variations of active sites and the structures. Obviously, the Fe single-atom densities of the different FeN_x_/C-*b* SACs in STEM images gradually increase without any metal clusters or nanoparticles (Supplementary Fig. 13), in accordance with the no observable Fe-Fe scattering path in FT-EXAFS spectra (Fig. 3b and Supplementary Fig. 14), which indicates that the confinement of the MOFs precursor is conducive to atomic dispersion. As shown in the Mössbauer spectra of FeN_x_/C-*b* SACs, which are derived from the recoil-free absorption of γ rays by ^57^Fe nuclei and can be deconvoluted into three different doublets (D1, D2, D3) according to the isomer shift (IS, *δ*_*iso*_) and quadrupole splitting (QS, *ΔE*_*Q*_) values^38–40^, indicating that the multiple FeN_x_ coordination configurations coexist in SACs, respectively corresponding to the square-planar FeN_4_, octahedral FeN_6_ and pyramidal FeN_5_ species (Fig. 3c). The asymmetrically axial N coordinate atom from the plane underneath the FeN_4_ moiety results in the out-of-plane coordination of FeN_5_ structure^18^.

Then, we measured the ensemble oxidase-like activity of FeN_x_/C-*b* SACs. The oxidase-like catalytic reaction is conducted in air-saturated sodium acetate–acetic acid buffer with optimized pH^41^. The oxidase-like activity can be parallelly determined by colorimetric assays of the UV-vis absorbance and fluorescence spectra due to the characteristic UV-vis absorbance and fluorescence emission peaks of the oxidative DAP at 450 nm and 565 nm, respectively (Supplementary Fig. 15). Meanwhile, the consistent trends of the activity changes in the two spectra along with the variation of the Fe atom loading have demonstrated the effectiveness and reliability of this oxidase-like catalytic reaction scheme both in the bulk and single-molecule fluorescence modes. The similar Michaelis-Menten curves of FeN_x_/C-*b* SACs also indicate their inherently similar kinetics and catalytic conditions (Supplementary Fig. 16).

According to the SAC-FCS method, the single-atom catalytic sites of FeN_x_/C-*b* SACs can be divided into two categories, FeN_5_ and FeN_4/6_, for the relatively active difference in oxidase-like catalysis. The FeN_5_ sites are defined as the more active single-atom sites (*E*_1_). In the same way, we measured the fluorescent trajectory of FeN_x_/C-*b* SACs at single-turnover resolution and calculated the catalytic behavior and kinetic parameters of the atomic active centers through the autocorrelation spectrum and the derived fitting equation. As shown in Fig. 3d, the series of FeN_x_/C-*b* SACs exhibit a similar kinetic process observed from autocorrelation spectra. Through the systematic SAC-FCS analysis of FeN_x_/C-*b* SACs, we find that the fitting curve with the terms of the free fluorescent diffusion and the oxidase-like reaction matched well with the autocorrelation data suggests that the calculations are close to the actual reaction kinetics (Supplementary Fig. 17). The statistic proportion of *E*_*1*_ in FeN_x_/C-*b* SACs exhibits a distinct Gaussian-like distribution and is determined to be 26.5 ± 1.2% after multipoint statistics of the fluorescent trajectories from different illuminated regions (Supplementary Fig. 18). According to the distribution of the FeN_5_/Fe atomic proportion of different SACs, there is no obvious change of the ratio of actual active sites with the increase of the Fe atom loading in a certain range, the apparent activity, thereby, can be promoted to some degree by increasing the metal loading. These results are further verified by Mössbauer spectra of the series of FeN_x_/C-*b* SACs. Notably, the D3 quadrupole doublets in different SACs, with similar *δ*_*iso*_, *ΔE*_*Q*_ and peak ratios (Supplementary Table 1), are consistent with the fitting results of active sites from SAC-FCS method (Fig. 3e).

**Fig. 4:**
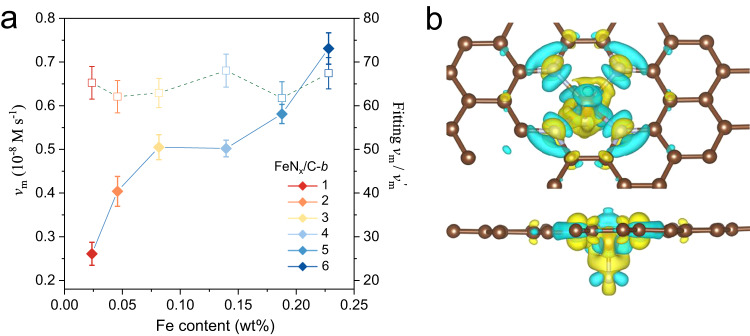
Single-atom kinetic and mechanism analysis of FeN_x_/C SACs. **a** SAC-FCS fitting single-atom *v*_m_ ratio (*v*_m_[FeN_5_]/*v*_m_^’^[FeN_4/6_], denoted in dashed line) and oxidase-like activity (denoted in solid line) of FeN_x_/C-*b* SACs toward the different Fe atomic loading. **b** DFT calculation of charge density difference plots for the FeN_5_ active sites with top view (upper) and front view (lower). The blue areas show where the electron density has been enriched, while the yellow areas show where the electron density has been depleted.

Meanwhile, we extrapolated the relative kinetic rates of the active sites in SACs from the autocorrelative fitting equation. As shown in Fig. 4a, there is a similar trend with the active site ratios. Both of them are not subject to the regulation of Fe contents. The average specific value of the *v*_m_/*v*_m_’ is about 65 (Supplementary Fig. 19); in consequence, the catalytic rate of the FeN_5_ center is much higher than that of the FeN_4/6_ sites. The orders of the magnitude dynamical difference indicate that the intrinsic active sites contribute more than 95% of the apparent oxidase-like activity with less atomic proportion in the FeN_x_/C SACs system.

By identifying the significant kinetic parameters of the *r*, *v*_m_, and *K*_m_ (Supplementary Fig. 20), we can quantify the inherent oxidase-like activity of each single-atom site from integral kinetic measurements and fluorescence spectroscopy. Then, we can achieve the equipotent comparison of the catalytic activity between SACs and natural enzymes at single atomic level. Theoretically, the oxygen reduction reaction is the rate-determining step in this enzyme-like redox catalysis; the favorable mechanism on the FeN_5_ site is illustrated by density functional theory calculation^16,18^. As depicted in the charge density difference of FeN_x_/C atomic centers (Supplementary Fig. 21), the asymmetrical FeN_5_ coordination structure markedly changes the electronic structure and stretches the Fe-N bonds^19,39,42^, which enhances the Fe-N charge transfer and activates the oxygen molecules via the electron push effect (Fig. 4b). Meanwhile, the partial density of states (PDOS) of the central Fe site revealed a negative shift of the *d*-band center in FeN_5_ (Supplementary Fig. 22), which will give rise to the weaker adsorption strengths of oxygenated intermediates (*OOH*, *O and *OH) on FeN_5_ site, and the higher oxygen reduction activity^43,44^. As a result, the unit oxidase-like activity (*K*_cat_) of the FeN_5_ single-atom site is quantified as about 3.92 s^−1^ in FeN_x_/C SACs (Supplementary Fig. 23), which is 182 times higher than that of FeN_4/6_ single-atom sites (0.0215 s^−1^) in FeN_x_/C SACs, and 1463 times higher than that of FeN_4_ single-atom sites (0.00268 s^−1^) in FePc (Supplementary Table 2). Then, we assessed the oxidase-like activity of FeN_x_/C SACs in catalyzing C–H bond selective oxidation. Ethylbenzene is selected as the reactant and oxygen as the oxidant. Gas chromatography–mass spectrometry (GC-MS) is employed to quantify the catalytic conversion and selectivity of ethylbenzene. Consistently, the as-prepared FeN_x_/C SACs exhibit much higher catalytic velocity and term turnover frequency than other catalysts (Supplementary Fig. 24a and Supplementary Table 3). The recycling experiments of FeN_x_/C SACs reveal that even after undergoing five cycles, there is no noticeable decline in the conversion and selectivity, indicating the remarkable stability of the catalysts (Supplementary Fig. 24b). These reveal the inherent coordination-dependent catalysis and ultrahigh oxidase-like activity of FeN_5_ site in FeN_x_/C SACs, as well as the non-negligible factor of the unidentical single-atom sites in SACs. Similarly, identifying and quantifying the real active sites in SACs will have great significance and can facilitate the related electrocatalysis and heterogeneous catalysis as well^19,21^.

## Discussion

To summarize, we develop a quantitative SAC-FCS method consisting of the single-atom catalyst models, heterogeneous redox reaction schemes, single-molecule measurements and autocorrelation analysis for identifying and resolving the inherent active sites of Fe-N SACs toward oxidase-like catalysis. As a result, the SAC-FCS method is successfully applied to two categories of unequal FeN_x_ SACs models with twenty-four continuous-variable groups and uncovered their important atomic kinetics of the active sites. Specifically, the proportion of the FeN_5_ active site in FeN_x_/C SACs is approximately 26%, and the catalytic rate constants of the active site toward oxidase-like catalytic reaction could be 182- and 1463-times higher than that of the inactive sites in FeN_x_/C SACs and the FeN_4_ site in iron phthalocyanine, respectively. It should be noted that the single-atom kinetics of active site were tens of times the difference compared to that of the ensemble-averaged measurements. While the SAC-FCS method has limitations, such as the relative low resolution and sensitivity to different categories of single-atom sites, as well as simplified reaction and catalyst models, the accuracy can be improved by utilizing more universal probe molecules and optimizing the autocorrelation analysis. Through this study, we demonstrate the powerful complementarity and significance of this quantitative method for SACs technology toward the understanding, rational design and mechanism study of the single-atom active sites.

## Methods

### Immobilization of single-atom sites

The uniform immobilization of the models of the single-site catalysts on microscope cover slides was achieved by spin-coating of the metal phthalocyanine or FeN_x_/C SACs (detailed synthesis, characterization and analysis of the catalysts are in Supplementary Information 3.2). For metal phthalocyanine models, the diluted FePc, CoPc and NiPc were mixed in a fixed proportion; the proportion of FePc was 10%, 20%,…, 90%, successively. The measurement was performed on the cover slides in 100 mM of sodium acetate buffer with 1 nM of OPD. The time of each measurement was 2 min, and the real-time trace was obtained from the single-photon counter. The intensity time trace was obtained with the time bins 0.5 ms. The fluorescence signals obtained were stochastic in the single-molecule catalytic reaction. Meanwhile, there was a clear distinction between the signals where the single molecule reaction occurs in the observed region and the diffusion background and the fluorescence burst corresponding to the enzyme reaction.

### Measurement conditions

The SAC-FCS experimental setup was developed based on the microscope host (Carl Zeiss Observe A1). The laser beam from an ion argon laser (488 nm, 8 mW) would first pass a neutral density filter; the laser power was decreased to about 100 μW at the objective lens during data acquisition. The excitation light was reflected by a dichroic mirror into the high numerical aperture (NA, 1.45) oil-immersion objective (100 × Carl Zeiss). The fluorescence of the product was collected by the same objective, transmitted through the dichroic mirror, then reflected by a mirror and focused onto a pinhole (50 μm diameter). A blocking filter removed the reflected laser light at 488 nm. Thus, all the fluorescence signals were from the fluorescent product molecules (DAP) in the illuminated region. The fluorescence photons were detected by an avalanche photodiode (APD) (SPCM-AQR-, PerkinElmer). A photon counter (Pico Harp 300) was used to record the arrival time of each single photon. The autocorrelations were calculated directly from the arrival times of the detected photons. When performing the SAC-FCS measurement, the laser power was slightly adjusted according to the corresponding sample, which kept the average fluorescence intensity at about 5 × 10^4^ Counts/s. Using this fluorescence intensity measuring range, the fluorescence autocorrelation spectra were calculated efficiently. If the average fluorescence intensity was lower, it was difficult to get a stable correlation curve. If the average fluorescence intensity was too high, the triplet state dynamics would have an impact on the autocorrelation spectra of the single-atom catalysis reaction. Notice that the lagging time scale for the impacts from the triplet state was roughly from 1 × 10^−7^ to 1 × 10^−6^ s (detailed formula derivation is in Supplementary Information).

### Supplementary information

Supplementary Information

## Data Availability

The data that support the findings of this study are available from the corresponding authors upon reasonable request.
