# Supplementary material for: Identifying the active sites in unequal iron-nitrogen single-atom catalysts
Source: Nat Commun. 2023 Sep 11;14:5594. doi: 10.1038/s41467-023-41311-9 (PMC10495408; doi:10.1038/s41467-023-41311-9)
Supplement: Supplementary file 1 — Supplementary Information [file 41467_2023_41311_MOESM1_ESM.pdf]

Supplementary information for

Identifying the active sites in unequal iron-nitrogen single-atom catalysts

Liang Huang, Qiong Liu, Weiwei Wu, Ge Gao, Xiliang Zheng, Jin Wang*, Shaojun Dong*

*Corresponding author. Email: dongsj@ciac.ac.cn (S.D.), jin.wang.1@stonybrook.edu (J.W.)

The supplementary file includes:

1. Materials and Methods

1.1 Materials

1.2 Apparatus and characterization

1.3 Synthesis of single-atom catalyst models

1.4 Selective oxidation of C−H bonds on FeN_x/C SACs

2. SAC-FCS method

2.1 Immobilization of single-atom sites

2.2 Measurement condition of SAC-FCS

2.3 Details of the Reaction Scheme

2.4 Autocorrelation spectrum calculation

2.5 Curve-fitting for FCS data

3. Supplementary Discussion of SAC-FCS catalytic models

3.1 Single-atom model and SAC-FCS calculation

3.2. Structural Characterization of FeN_x/C SACs

3.3 SAC-FCS analysis for FeN_x/C SACs

1. Materials and Methods

1.1 Materials

Zinc nitrate hexahydrate ($Zn(NO_3)_2·6H_2O$, 99%), N, N-dimethylacetamide (DMAC), lauric acid (LA) were purchased from XiLong SCIENTIFIC. Co., Ltd. 2,2′-Bipyridine-5,5′-dicarboxylic acid (H_2bpydc, 98%) was obtained from Jilin Chinese Academy of Sciences - Yanshen Technology Co., Ltd. Iron phthalocyanine (FePc) was purchased from Energy Chemical (Shanghai) Co., Ltd. Polyvinyl pyrrolidone (average mol wt 40,000) and Zinc diacetate ($Zn(Ac)_2$, 99.9%) were purchased from Sigma Aldrich. 2-methylimidazole ($C_4H_6N_2$, 98%), cobalt phthalocyanine (CoPc), copper phthalocyanine (CuPc), acetate (HAc, 99.7%) and Sodium acetate (NaAc, 99.5%) were obtained from Aladdin (Shanghai, China). All the chemicals were used without further purification. Ultrapure water (≥18.2 MΩ cm) was used throughout the study.

1.2 Apparatus and characterization

The morphologies of the single-molecule and single-atom samples were characterized by transmission electron microscope (TEM) and scanning electron microscope (SEM). SEM images were collected via a Zeiss Gemini Sigma 300 SEM instrument. TEM images were obtained with a HITACHI 600 TEM operated at 100 kV. The high-resolution TEM (HRTEM) and high-angular annular dark-field (HAADF) scanning TEM (STEM) imaging were conducted using a probe aberration-corrected FEI Themis Z microscope at 300 keV accelerating voltage. The energy dispersive X-ray spectroscopy (EDS) mapping was acquired using FEI Themis Z microscope equipped with a FEI Super-X™ detector system.

The atomic composition and structure of the samples were characterized by X-ray spectra. X-ray photoelectron spectroscopy (XPS) measurements were performed on an ESCALABMKII (VG Co., UK) spectrometer with an Al Kα excitation source. Powder X-ray diffraction (XRD) patterns of samples were recorded using a D8 ADVANCE (Bruker, Germany) diffractometer with Cu K radiation (λ=1.54 Å). X-ray absorption spectroscopy (XAS) was measured at the beamline 1W1B station of the Beijing Synchrotron Radiation Facility, China. The X-ray absorption fine structure (XAFS) and X-ray absorption near-edge structure (XANES) spectra of the Fe *K*-edge were recorded in a fluorescence mode, with Fe foil, Fe_2O_3, Hemin and FePc as references. The storage ring was working at the energy of 2.5 GeV with an average electron current of 250 mA. The hard X-ray was monochromatized with Si (111) double-crystals. The acquired EXAFS data were extracted and processed according to the standard procedures using the ATHENA module implemented in the IFEFFIT

software packages. The k^3-weighted EXAFS spectra were obtained by subtracting the post-edge background from the overall absorption and then normalizing with respect to the edge-jump step. Subsequently, k^3-weighted $\chi(k)$ data in the k-space were Fourier-transformed to real (R) space using a hanning windows (d_K = 1.0 $Å^{-1}$) to separate the EXAFS contributions from different coordination shells. The ^{57}Fe Mössbauer spectrum was carried out on a Topologic 500A spectrometer driving with a proportional counter at room temperature. The radioactive source was ^{57}Co (Rh) moving in a constant acceleration mode. The spectra were fitted individually with the appropriate combinations of Lorentzian lines using the program MossWinn 4.0, which integrated the database management and data analysis functionalities. The components of iron phases were identified based on their Mössbauer parameters including the isomer shift (δ_{iso}), quadruple splitting (ΔE_Q) and magnetic hyperfine field, and relative area of Fe ions.

The Fe loadings of the single-atom catalysts were measured by inductively coupled plasma-mass spectrometry (ICP-MS) analysis, which were obtained by an Agilent 7700x (Agilent Technologies). The nitrogen adsorption/desorption isotherms were obtained with an ASAP 2020 Physisorption Analyzer (Micrometrics Instrument Corporation). UV-vis absorption measurements were carried out on an Agilent Cary 60 (Varian) UV-vis-near-infrared (NIR) spectrometer. Fluorescence measurements were conducted with an Agilent Cary Eclipse fluorescence spectrophotometer. Fourier transform infrared (FT-IR) spectra were obtained using a VERTEX 70 FT-IR spectrophotometer (Bruker, Germany).

Computational methods. All periodic DFT calculations were carried out using the Vienna ab initio simulation package (VASP, version 5.4.4)[1-3], which approximately describes the ionic cores according to the projector augmented wave (PAW) method and evaluate the exchange and correlation energies using the Perdew-Burke-Ernzerhof (PBE) functional. The cutoff energy of electronic wave functions was set at 450 eV. The Gaussian electron smearing method with σ = 0.05 eV was applied during the calculations. Geometric optimization converged until the forces acting on the atoms were smaller than 0.01 eV $Å^{-1}$, whereas the energy threshold-defining self-consistency of the electron density was set to 10^{-5} eV. The catalytic models were constructed using an 8×8 graphene supercell, and a 15 Å vacuum layer was chosen to avoid the interaction between sheets in the periodic images. Three FeN_x/C configurations in graphene with pyridine N and pyrrolic N were considered and fully optimized.

1.3 Synthesis of FeN_x/C SACs-*b* catalyst models

Firstly, the leaf-like zeolitic imidazolate framework (ZIF-L) nanosheets were prepared according to the literatures previously reported[4]. 0.744 g of $Zn(NO_3)_2·6H_2O$ and 1.642 g of 2-methylimidazole were dissolved in 50 mL of H_2O separately. Then mixed the above two solutions and kept stirring for 4 h at 30 °C. The products were washed with water for five times, collected by centrifugation and dried at 60 °C for 8 h. Secondly, FePc@Zn-MOFs were prepared through the reconstruction of the ZIF-L. Briefly, 50 mg ZIF-L nanosheets, 40 mg of PVP, 50 mg of H_2bpydc, 100 mg of LA, 50 mg of $Zn(Ac)_2$ and different amounts of FePc were successively added into 30 mL of DMAC and then treated with ultrasonic. The resulting suspensions were transferred to 50 mL round-bottom flask and kept refluxing at 100 °C for 12 h under continuous stirring. The products were washed with DMAC and ethanol for five times, collected by centrifugation and dried at 60 °C for 8 h. Finally, the FeN_x/C single-atom catalysts (SACs) were prepared by pyrolysis of the MOFs precursors. The obtained FePc@Zn-MOFs were placed in a tube furnace at 900 °C for 2 h under N_2 condition, with a heating rate of 5 °C/min. After cooling to room temperature, the obtained solids were sonicated with 1 M hydrochloric acid for 4 h to remove the possible nanoparticles, washed with water for five times and dried at 60 °C in vacuum oven for 8 h to obtain FeN_x/C SACs-*b* catalysts, b = 1, 2, 3, 4, 5, 6, corresponding to the final Fe loading of 0.0237, 0.0458, 0.0815, 0.1395, 0.1877 and 0.228%, respectively.

1.4 Selective oxidation of C−H bonds on FeN_x/C SACs

Typically, 20 mg of the as-prepared FeN_x/C-3 SACs catalysts were homogeneously dispersed in 2.0 mL of glacial acetic acid solvent (Sigma-Aldrich, 100%) using an ultrasonic cleaner. Subsequently, 1.0 mmol of ethylbenzene substrate (Sigma-Aldrich, 99.8%) was added to the mixture and stirred in a 10 mL quartz-lined stainless-steel autoclave equipped with a magnetic stirrer. Once the reactor was sealed, the air was evacuated by O_2 flushing and pressurized to 0.3 MPa. The reaction was conducted for 12 hours at 130 °C under stirring. After cooling the reactor to room temperature, the catalysts were filtered and recovered, and the filtrate was subjected to extraction using 8 mL of ethyl acetate (Sigma-Aldrich, 99.9%). The resulting products were analyzed and quantified with the external standard method using gas chromatograph-mass spectrometer (GC-MS, Agilent 5977A). The steady-state kinetics parameters were obtained by measuring the change of the initial velocity (v) with the concentration of the substrate. The Michaelis-Menten constants (K_m) and maximum initial velocity (V_m) were obtained by fitting with Michaelis-Menten equation $v=V_m·[S]/(K_m+[S])$. The catalytic rate constant (k_{cat}) was obtained by $k_{cat}=V_m/[E]$, where [E] is the concentration of the single-atom sites[5].

2. SAC-FCS method

2.1 Immobilization of single-atom sites

The uniform immobilization of the models of the single-site catalysts on microscope cover slides was achieved by spin-coating of the metal phthalocyanine or FeN_x/C SACs. For metal phthalocyanine models, the diluted FePc, CoPc and NiPc were mixed in a fixed proportion, the proportion of FePc was 10%, 20%, …, 90%, successively. The measurement was performed on the cover slides in 100 mM of sodium acetate buffer, with 1 nM of OPD. The time of each measurement was 2 minutes, and the real time trace was obtained from the single-photon counter. The intensity time trace was obtained with the time bins 0.5 ms. The fluorescence signals obtained were stochastic in the single molecule catalytic reaction. Meanwhile, there was a clear distinction between the signals where the single molecule reaction occurs in the observed region and the diffusion background, and the fluorescence burst corresponding to the enzyme reaction.

2.2 Measurement condition of SAC-FCS

The SAC-FCS experimental setup was developed base on the microscope host (Carl Zeiss Observe A1). The laser beam from an ion argon laser (488 nm, 8 mW) would first pass a neutral density filter, the laser power was decreased to about 100 μW at the objective lens during data acquisition. The excitation light was reflected by a dichroic mirror into the high numerical aperture (NA, 1.45) oil-immersion objective (100 × Carl Zeiss). The fluorescence of the product was collected by the same objective, transmitted through the dichroic mirror, then reflected by a mirror and focused onto a pinhole (50 μm diameter). A blocking filter removed the reflected laser light at 488 nm. Thus, all the fluorescence signals were from the fluorescent product molecules (DAP) in the illuminated region. The fluorescence photons were detected by an avalanche photodiode (APD) (SPCM-AQR-, PerkinElmer). A photon counter (Pico Harp 300) was used to record the arrival time of each single photon. The autocorrelations were calculated directly from the arrival times of the detected photons. When performing the SAC-FCS measurement, the laser power was slightly adjusted according to the corresponding sample, which kept the average fluorescence intensity at about 5×10^4 Counts/s. Using this fluorescence intensity measuring range, the fluorescence autocorrelation spectra were calculated efficiently. If the average fluorescence intensity was lower, it was difficult to get a stable correlation curve. If the average fluorescence intensity was too high, the triplet state dynamics would have an impact on the autocorrelation spectra of the single-atom catalysis reaction. Notice that the lagging time scale for the impacts from triplet state was roughly from 1×10^{-7}

to 1 × 10^{-6} s.

2.3 Details of the reaction scheme and autocorrelation

The kinetic processes of the different single-atom sites were observed by fluorescence correlation spectra during the oxidase-like catalysis reaction of oxidizing the non-fluorescent substrate (OPD) into the fluorescent product (DAP). According to the single-atom catalytic mechanism, the coordination structure of the central atom of single site catalysts significantly affects the intrinsic catalytic property. Normally, the atomic activity of the real active site is several times to hundreds of times than that of the other atomic sites. Therefore, the single-atom sites can be simply identified as the faster and slower parts, which are denoted as E_1 and E_2, respectively. Then the measuring system contains two kinds of catalytic kinetics processes, the kinetics of the reaction system are expressed by the following kinetic equations:

$$\text{o}-\text{phenylenediamine (OPD)}+O_2\xrightarrow{FeN_x}2,3-\text{diaminophenazine (DAP)}$$

$$E_1+S\underset{k_{-1}}{\overset{k_1[S]}{\rightleftharpoons}}E_1S\xrightarrow{k_2}E_1+P$$

$$E_2+S\underset{k_{-1}^{'}}{\overset{k_1^{'}[S]}{\rightleftharpoons}}E_2S\xrightarrow{k_2^{'}}E_2+P$$

Take the E_1 as an example, the stochastic kinetics of single-atom site E_1 are described as follows:

$$\frac{d[E]}{dt}=-k_1[E][S]+[ES]k_{-1} \qquad (S1)$$

$$\frac{d[ES]}{dt}=k_1[E][S]-[ES](k_2+k_{-1}) \qquad (S2)$$

$$\frac{d[P]}{dt}=k_2[ES] \qquad (S3)$$

The probability distribution for the waiting time also can be calculated as the equation:

$$f(t)=\frac{d[P]}{dt}=k_2P_{ES}(t) \qquad (S4)$$

$$=\frac{\lambda_1\lambda_2}{\lambda_2-\lambda_1}\left(e^{-\lambda_1 t}-e^{-\lambda_2 t}\right) \qquad (S5)$$

Where $\lambda_{1,2}$ is the eigenvalue of *Equation S1*, $\lambda_{1,2}=\frac{1}{2}(k_1[S]+k_{-1}+k_2\pm\sqrt{(k_1[S]+k_{-1}+k_2)^2-4k_1k_2[S]})$, and *f(t)* satisfies the normalization condition $\int_0^\infty f(t)dt=1$. Through few calculations, it is easy to verify that the mean waiting time, $<T>=(\lambda_1+\lambda_2)/(\lambda_1\lambda_2)$. The derivation of reaction sites E_2 can be done in the same way.

In a turnover experiment, only one fluorescent product is monitored at a time, regardless of the production process of the fluorescent product. The time for the completion of the next catalytic reaction is a stochastic variable that can be

completely characterized by a waiting time distribution *f(t)*.

$$f(t) = \frac{d[P]}{dt} = k_2' P_{ES}(t) \quad (S6)$$

$$= \frac{\lambda_1' \lambda_2'}{\lambda_2' - \lambda_1'} (e^{-\lambda_1' t} - e^{-\lambda_2' t}) \quad (S7)$$

And the mean waiting time is $< T >= (\lambda_1' + \lambda_2')/(\lambda_1' \lambda_2')$. The above derivation is for a single atom catalysis of the SACs in the observation region. When there are not just one but rather a few copies of the single-atom sites in the measurement area of SAC-FCS, the kinetics need to include the stochastic numbers of catalytic sites in the states *E* and *ES*, etc. Let there be *m* identical and randomly pick a time, and let T^{ss} be the waiting time for the next arrival of the product, then T^{ss} is known as stationary residual time in renewal theory. The distribution is different from *f(t)* in *Equation S5*, the cumulative probability distribution of waiting time when another product molecule be observed is:

$$P\{T^{SS} \leq t\} = \frac{\int_0^t P\{T \geq s\}}{< T >} = 1 - \frac{\lambda_2^2 e^{-\lambda_1 t} - \lambda_1^2 e^{-\lambda_2 t}}{\lambda_2^2 - \lambda_1^2} \quad (S8)$$

$$P\{T > s\} = \int_s^\infty f(t) dt \quad (S9)$$

$$= \frac{\lambda_1 \lambda_2}{\lambda_2 - \lambda_1} \left(\frac{e^{-\lambda_2 s}}{\lambda_2} - \frac{e^{-\lambda_1 s}}{\lambda_1} \right) \quad (S10)$$

Then the integral of $\int_0^t P\{T \geq s\}$ can be calculated by a simple integral formula. The result of integration is shown in *Equation S8*. The same results can be obtained for catalytic active site E_2 as follow:

$$P\{T^{SS} \leq t\} = \frac{\int_0^t P\{T \geq s\}}{< T >} = 1 - \frac{{\lambda_2'}^2 e^{-\lambda_1' t} - {\lambda_1'}^2 e^{-\lambda_2' t}}{{\lambda_2'}^2 - {\lambda_1'}^2} \quad (S11)$$

Differentiating the cumulative probability distribution for the waiting time of the arrival of the next product molecule, the probability density function for the next product molecule arrival T^{ss} can be evaluated according to the following equation:

$$f_{T^{SS}}(t) = \frac{\lambda_1 \lambda_2}{\lambda_2^2 - \lambda_1^2} \left(\lambda_2 e^{-\lambda_1 t} - \lambda_1 e^{-\lambda_2 t} \right) \quad (S12)$$

$$f'_{T_{SS}}(t) = \frac{\lambda_1' \lambda_2'}{{\lambda_2'}^2 - {\lambda_1'}^2} \left(\lambda_2' e^{-\lambda_1' t} - \lambda_1' e^{-\lambda_2' t} \right) \quad (S13)$$

For the whole observation region, the cumulative probability distribution of the average residence time can be divided into two parts, one for the faster catalytic reaction process, the other for the slower catalytic reaction process. Suppose the number of the faster catalytic reaction process is m_1, and the number of slower catalytic reaction process is m_2 and $m_1 + m_2 = \mathrm{m}$, and define $m_1/m = r_1$ and $m_2/m = r_2$,

the cumulative probability distribution is

$$F(t) = 1 - \left(\int_t^{\infty} f\,(x)dx\right)\left(\int_t^{\infty} f_{T^{SS}}\,(x)dx\right)^{(m_1-1)} - \left(\int_t^{\infty} f'\,(x)dx\right)\left(\int_t^{\infty} f'_{T^{SS}}\,(x)dx\right)^{(m_2-1)} \quad (S14)$$

Here r_1, r_2 is the proportion of the single-atom active sites for the faster and slower catalytic reaction process, respectively, and $r_1 + r_2 = 1$. If we rescale $t = \tau/m$, in the limit of $m\rightarrow\infty$ we obtain the following equations:

$$F(t) = 1 - \left(\int_t^{\infty} f_{T^{SS}}\,(x)dx\right)^{(mr_1-1)} - \left(\int_t^{\infty} f'_{T^{SS}}\,(x)dx\right)^{(mr_2-1)} \quad (S15)$$

$$= 1 - \left(\frac{\lambda_2^2 e^{-\lambda_1 t} - \lambda_1^2 e^{-\lambda_2 t}}{\lambda_2^2 - \lambda_1^2}\right)^{(mr_1-1)} - \left(\frac{{\lambda'_2}^2 e^{-\lambda'_1 t} - {\lambda'_1}^2 e^{-\lambda'_2 t}}{{\lambda'_2}^2 - {\lambda'_1}^2}\right)^{(mr_2-1)} \quad (S16)$$

According to the condition m→∞, the exponent can be used Taylor expansion. *Equation S16* can be reduced to *Equation S17*. By inverting the first order Taylor expansion of the *equation*, the result is described by the *Equation S18:*

$$F(t) = 1 - \left(1 - \frac{\lambda_1\lambda_2\tau}{(\lambda_1+\lambda_2)m}\right)^{(mr_1-1)} - \left(1 - \frac{\lambda'_1\lambda'_2\tau}{(\lambda'_1+\lambda'_2)m}\right)^{(mr_2-1)} \quad (S17)$$

$$= 1 - e^{-\frac{\lambda_1\lambda_2}{\lambda_1+\lambda_2}\tau r_1} - e^{-\frac{\lambda'_1\lambda'_2}{\lambda'_1+\lambda'_2}\tau r_2} \quad (S18)$$

Thus, the probability density of the waiting time of the next catalytic reaction is:

$$f(t) = \frac{dF(t)}{dt} = \frac{\lambda_1\lambda_2 r_1}{\lambda_1+\lambda_2} e^{-\frac{\lambda_1\lambda_2}{\lambda_1+\lambda_2}t r_1} + \frac{\lambda'_1\lambda'_2 r_2}{\lambda'_1+\lambda'_2} e^{-\frac{\lambda'_1\lambda'_2}{\lambda'_{1_1}+\lambda'_2}t r_2} \quad (S19)$$

Due to the inevitable diffusion of the fluorescent molecules in the solution, the diffusion effect needed to be considered in the autocorrelations. Therefore, the final form of the autocorrelation can be rewritten as:

$$G(\tau) = c_1 e^{-r_1 V_1 \tau} + c_2 e^{-r_2 V_2 \tau} + \frac{1}{N}\left[1 + \frac{\tau}{\tau_D}\right]^{-1}\left[1 + \frac{\tau}{\tau_D}\omega^2\right]^{-\frac{1}{2}} + c_3 \quad (S20)$$

Here, τ_D is the diffusion time of the free fluorescent molecules and depends on the diffusion coefficient, N is the mean number of the fluorescent molecules in the observation region, and ω is the aspect ratio of the radius and the depth of the laser point, which is a constant during the fitting process and determined by the standard rhodamine 6G sample. V_1 and V_2 are the single-site reaction rates of the faster and slower oxidase-like catalytic processes, respectively. c_1, c_2, and c_3 are the fitting parameters depending the catalytic reaction sites. The first three items in *Equation S20* are the autocorrelation equations of faster reaction process, slower reaction process and the free diffusion process, respectively, the last one of c_3 is the baseline of

the autocorrelation. By comparing the first two items of *Equation S20* with *S19*, and considering $V = (\lambda_1\lambda_2)/(\lambda_1 + \lambda_2) = V_m[S]/(K_m + [S])$, we can obtain the following relationships:

$$c_1 = Kr_1\frac{V_m}{K_m} \quad (S21)$$

$$c_2 = Kr_2\frac{V'_m}{K'_m} \quad (S22)$$

$$V_1 = \frac{V_m[S]}{K_m + [S]} \quad (S23)$$

$$V_2 = \frac{V'_m[S]}{K'_m + [S]} \quad (S24)$$

$$r_1 + r_2 = 1 \quad (S25)$$

Therefore, the established autocorrelation *Equation S20* can be used to fit the experimental curves, meanwhile, the kinetic parameters of the different single-atom sites in the observation region can be solved by the restricted condition *Equation S21*-23.

2.4 Autocorrelation spectrum calculation

According to the SAC-FCS method, the fluorescence signals are the fluorescent product of DAP, which was stochastically obtained from the single-atom catalytic reactions. The fluorescence autocorrelation spectrum was calculated through the single molecule fluorescence signals to quantify the kinetic process of the fluorescence product and atomic sites. Single molecule fluorescence signals are recorded by the arrival time for each photon from the fluorescence. The raw single molecule fluorescence arrival data is a sequence of the arrival time of single photons. The sequence is denoted as $\{t_1, t_2, \dots, t_N\}$, where N is the total number of detected photons during one measurement. The measurement time is 2 minutes. The detected time is the integer multiples of minimal time $\delta t = 4 \times 10^{-12}$ s determined by the temporal resolution the single photon counter (Pico Harp 300). Thus, the absolute detection time is $\{t_1, t_2, \dots, t_N\}$ multiples δt. The lag time is needed to be determined before calculating the autocorrelation spectrum. The lag time of autocorrelation spectrum is not all possible values of lag time, because the possible lag time values are quite a lot. It is not necessary to calculated all possible lag time values since relatively few values are enough to characterize the shape of the autocorrelation curve. Therefore, the lag time is selected in the logarithmic space. For a given lag time τ in the autocorrelation spectrum, the algorithm starts with the original vector t_1, and check whether $t_1 + \tau$ is in the detection vector sequence $\{t_1, t_2, \dots, t_N\}$. If $t_1 + \tau$ is in the detection vector, the cumulative value is increased by one. Then one moves to the next detection time t_2, iterate until to the end of the detection vector. The algorithm

goes back to the original detection vector for anther lag time. After that the cumulative values of corresponding lag time are divided by the total number of summed intervals during one measurement, and the autocorrelation spectrum is obtained.

2.5 Curve-fitting for FCS data

The fitting function *Equation S20* includes a free diffusion part, which contains two parameters related to the size of observation space, the width and depth of illumination volume. These two parameters are measured by standard Rhodamine 6G fluorescent molecular diffusion. Rhodamine 6G is a fluorescent organic dye that can be excited by 488 nm laser. Its diffusion coefficient is 4.1 × 10^{-10} m^2s^{-1} in water at 25 ℃, and we use Rhodamine 6G to determine the illumination volume. The width and depth of the spot are obtained by the fitting to the autocorrelation of the fluorescence for free diffusion of the standard Rhodamine 6G molecules. The fitting function is:

$$G(\tau) = \frac{1}{N}\left[1+\frac{\tau}{\tau_D}\right]^{-1}\left[1+\frac{\tau}{\tau_D}\omega^2\right]^{-\frac{1}{2}} \qquad (26)$$

Where $\omega = r_0/z_0$, the fit parameters τ_D = 96 μs and ω = 0.6552. From these data, we obtained that r_0 = 0.19 μm and z_0 = 0.29 μm. Since the observation volume is determined mainly by the laser profiles and the equipment, it is expected to be the same for all the sample including Rhomine 6G and OPD. The geometrical parameters are held fixed in the SAC-FCS analysis of the FeN_x and *FePc/CoPc* single-atom models.

3. Supplementary discussion of SAC-FCS catalytic model

3.1 Single-atom model and SAC-FCS calculation

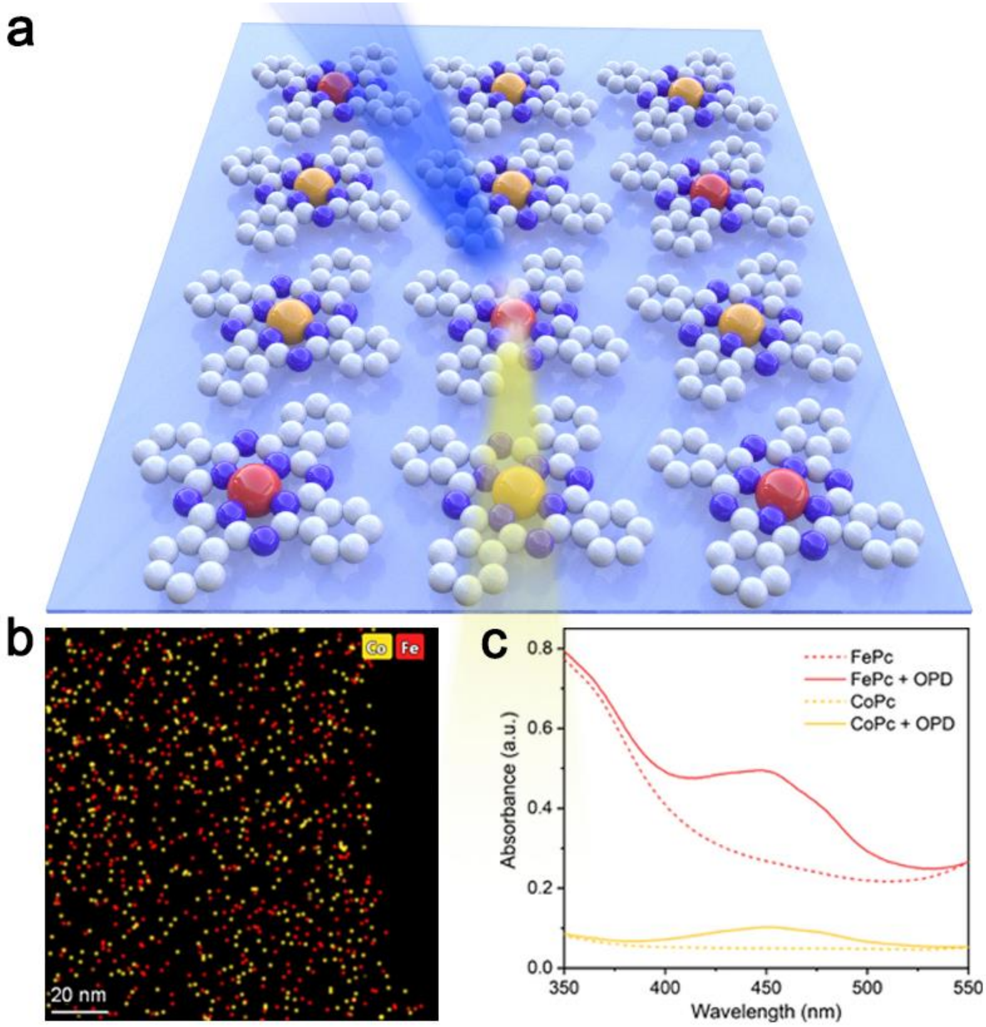

Supplementary Fig. 1 | Single-site metal phthalocyanine model. (a) Schematic illustration of the uniform immobilized FePc sites in the illuminated region of the microscope cover slide. The red, yellow, blue, and white balls respectively represent the Fe, Co, N and C atoms of the metal phthalocyanine. (b) Additional EDS mappings of the Fe and Co atomic distribution of the single-site metal phthalocyanine model. The red and yellow pixels represent the Fe and Co element signals, respectively. (c) UV-vis absorbance spectra of the catalytically oxidized OPD by FePc and CoPc in air-saturated buffer, respectively.

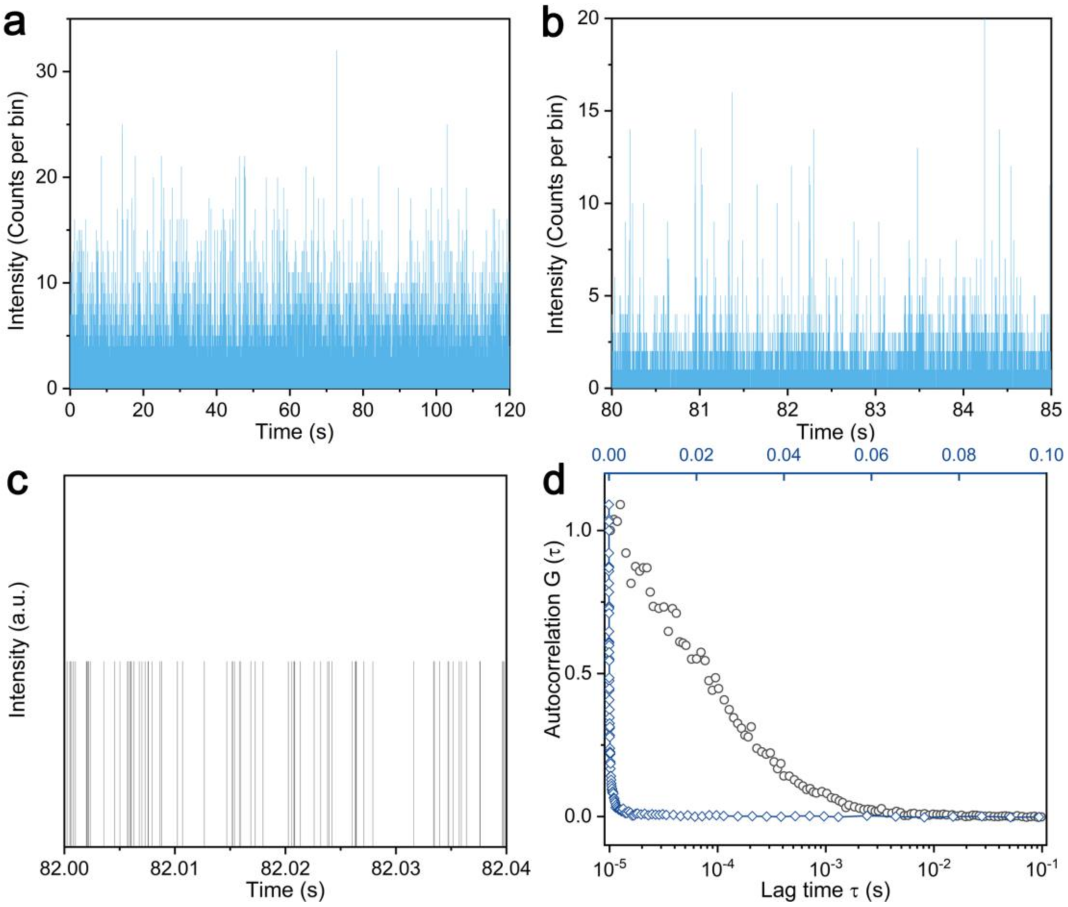

Supplementary Fig. 2 | Single-molecule fluorescence signals. (a) A typical and (b) partial fluorescence trajectory, (c) single-molecule fluorescence signals and (d) the experimental autocorrelation data of the FePc/CoPc-0.3 model collected from a random location in microflow cells with the substrates of the O_2 and 1 nM OPD.

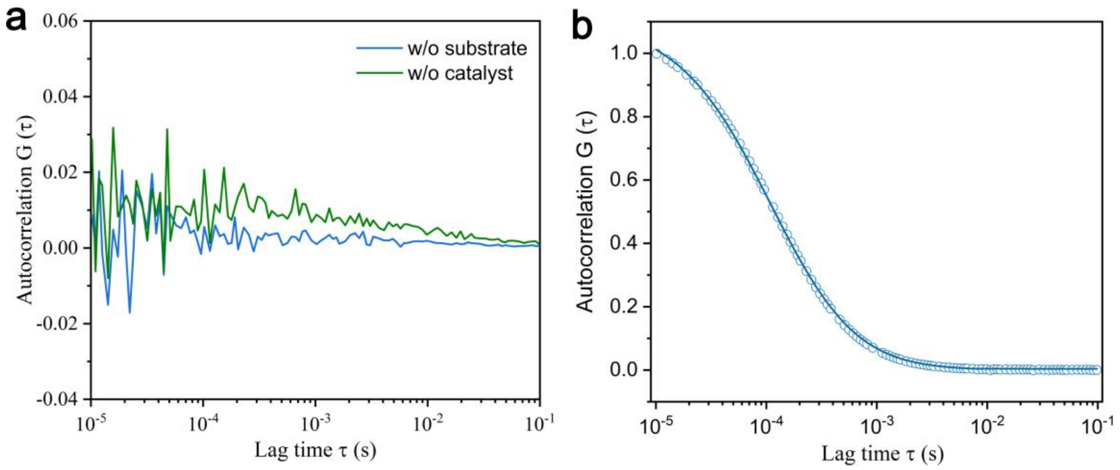

Supplementary Fig. 3 | Background of fluorescence correlation spectroscopy and autocorrelation. (a) Autocorrelation data obtained from 100 mM acetic buffer (pH 4.0) without the substrates or the catalysts. (b) The autocorrelation function for Brownian motion of Rhodamine 6G in water.

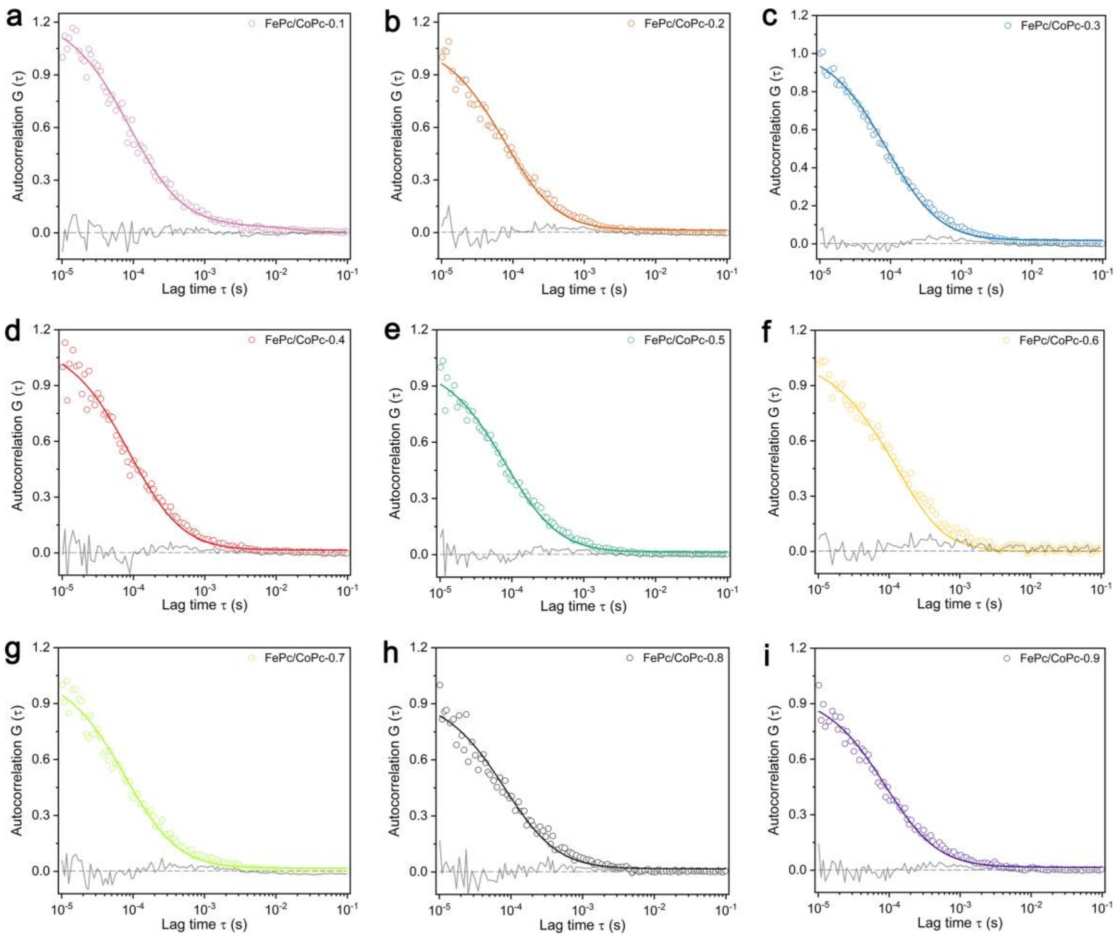

Supplementary Fig. 4 | Autocorrelation data and fitting curves of the binary FePc/CoPc model. (a-i) Typical fluorescence signals and autocorrelation of different FePc/CoPc-*a* catalytic models, respectively, *a* = 0.1, 0.2...0.9, which represents the actual ratio of FePc in the model. The circles represent the autocorrelation data from experiments and solid lines are fittings of *Equation 20* to the data, and gray lines are the corresponding errors of the fittings.

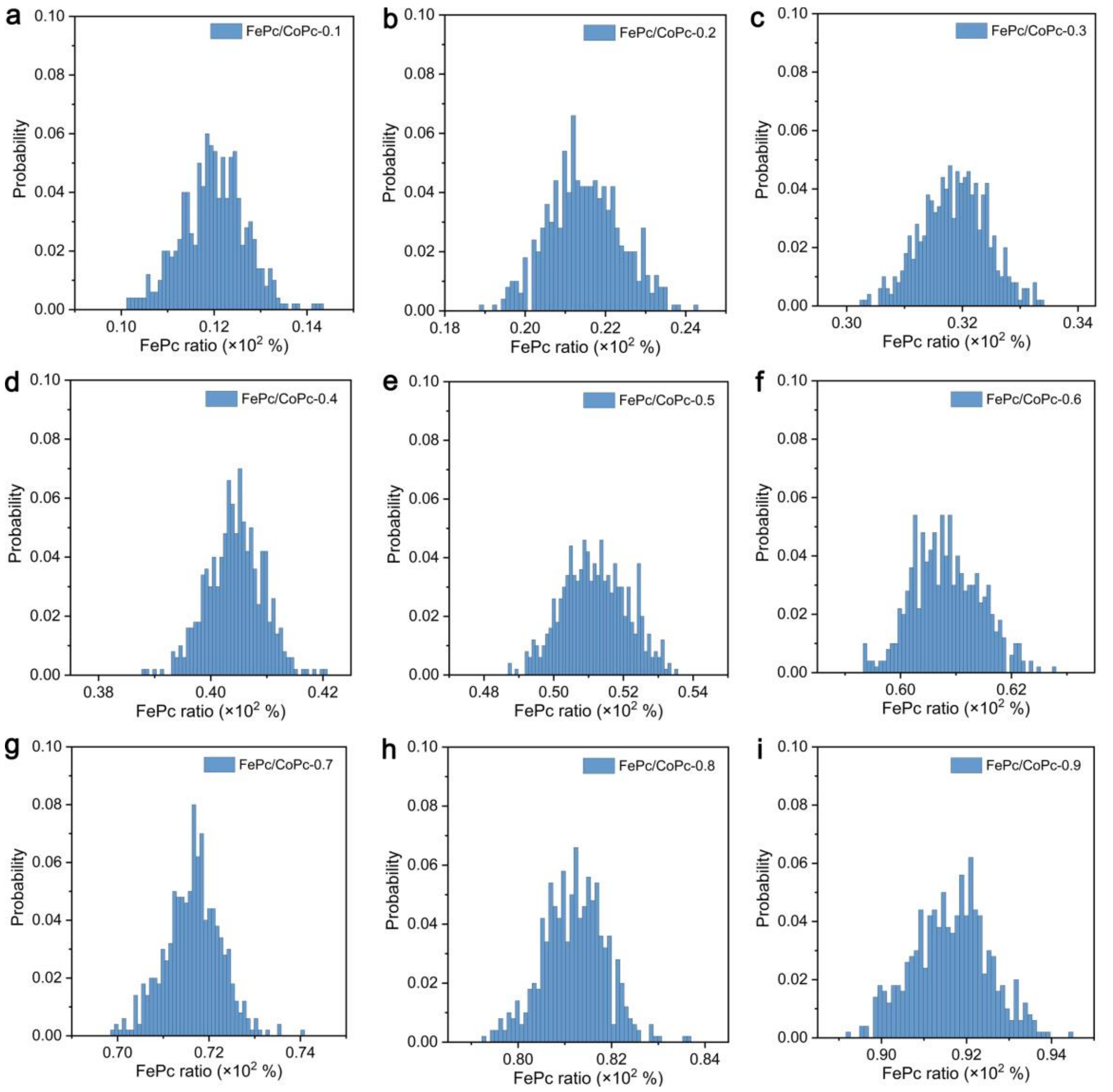

Supplementary Fig. 5 | SAC-FCS fitting ratio of FePc in FePc/CoPc model. (a-i) Intensity histogram of the fitting ratio of FePc sites in different FePc/CoPc-*a* catalytic models, where *a* represents the actual ratio of FePc in each model.

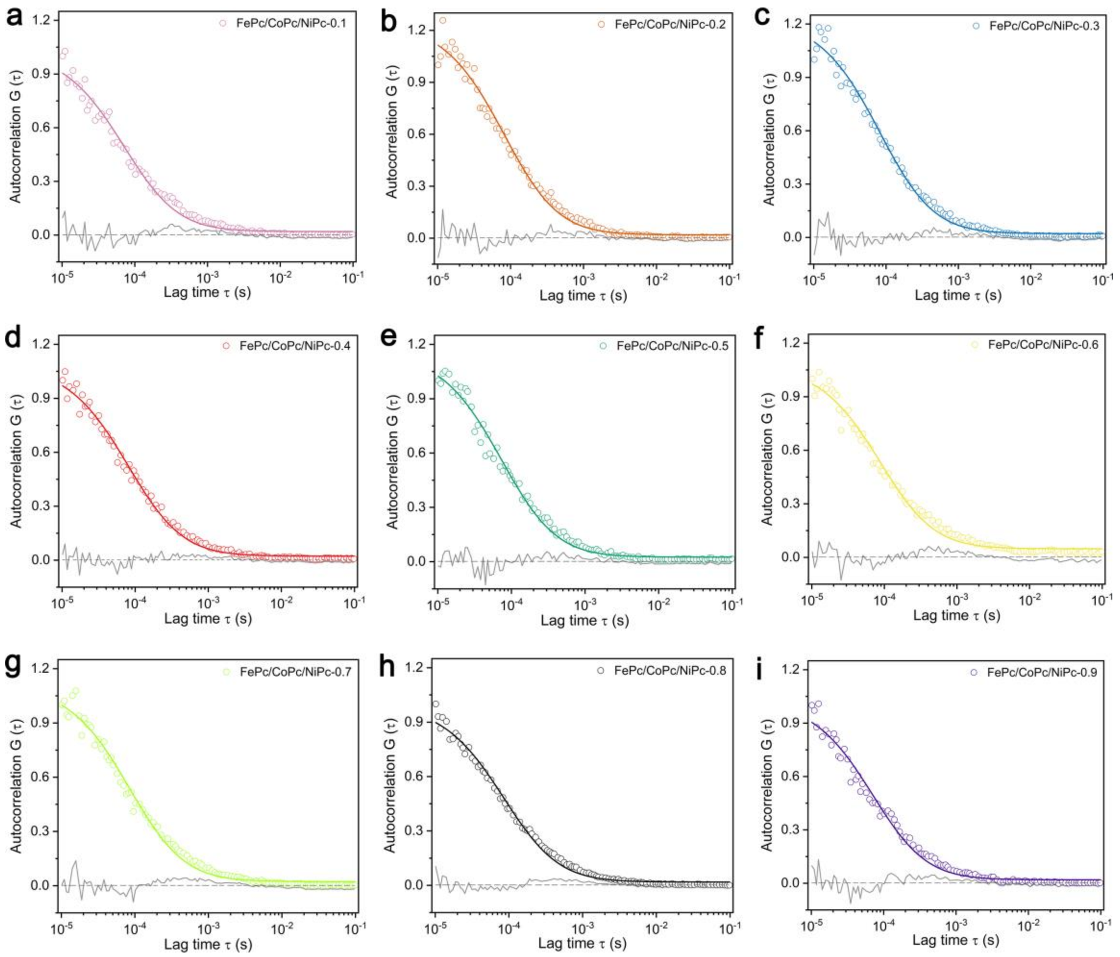

Supplementary Fig. 6 | Autocorrelation data and fitting curves of the ternary FePc/CoPc/NiPc model. (a-i) Typical fluorescence signals and autocorrelation of different FePc/CoPc/NiPc-*a* catalytic models, respectively, *a* = 0.1, 0.2...0.9, which represents the actual ratio of FePc in the model. The circles represent the autocorrelation data from experiments and solid lines are fittings of *Equation 20* to the data, and gray lines are the corresponding errors of the fittings.

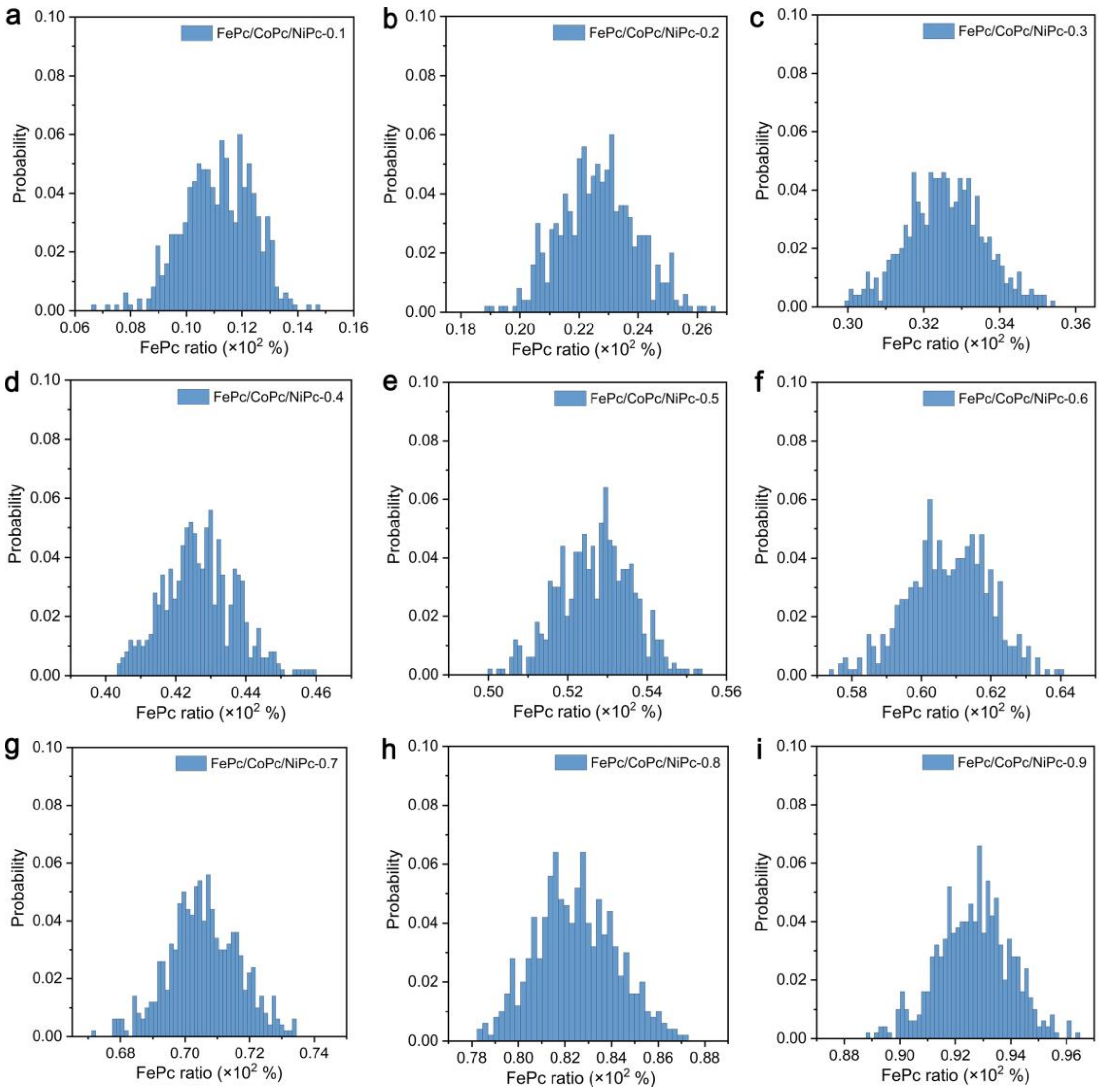

Supplementary Fig. 7 | SAC-FCS fitting ratio of FePc in FePc/CoPc/NiPc model. (a-i) Intensity histogram of the fitting ratio of the FePc sites in different FePc/CoPc-*a* catalytic models, where *a* represents the actual ratio of FePc in each model.

Supplementary Discussion 3.1

As shown in Supplementary Fig. 1, we first set up the single-atom catalyst model of the metal phthalocyanines by homogeneously dispersing these molecules over the nano-scanning stage. The significant differences of the metal phthalocyanines in catalytic activity and kinetics, which are derived from their symmetrical coordination structure and different central atoms, make them as the hybrid atomic sites. For instance, FePc exhibited much higher oxidase-like activity than that of the CoPc determined by the colorimetric assays. The absorbance of the 2,3-diaminophenazine (DAP) obviously increased with the presence of FePc under the standard oxidase-like catalysis. Meanwhile, the inherent catalytic rates of FePc and CoPc were also markedly different (Supplementary Fig. 1c). Therefore, the randomly dispersed metal phthalocyanine molecules in the observation region would compose the two-dimensional catalytic model that contains the different single-atom catalytic sites for the SAC-FCS analysis.

Attributing to the ultrahigh temporal resolution of the single photon counter (integer multiples of the minimal time $\delta_t = 4 \times 10^{-12}$s), we gathered every single molecule fluorescence signal produced by each atomically catalytic site through being converted to single photons, and distinguish them from the different arrival and interval time. As shown in Supplementary Fig. 2a-b, the raw arrival data of single molecule fluorescence was the turnover time traces of the atomic sites, which could be normalized and converted into a sequence of the arrival time of single photons (Supplementary Fig. 2c), and the interval time of two single photons was the time between two adjacent events. By selecting in the logarithmic space and iterating different lag time τ until to the end of the detection vector, we obtained the well-organized autocorrelation spectrum of the distribution of lag time (Supplementary Fig. 2d). Reversely, the autocorrelation curves at the position without catalysts or substrates showed negligible G(τ) signals with no apparent trends compared to the standard single-atom catalyst model (Supplementary Fig. 3), which indicated that the single molecule fluorescence and the autocorrelation were derived from the single-site catalysis rather than the background signal.

According to the autocorrelation equation of *Eq.* 20, including the parameters of the faster reaction process, slower reaction process, free diffusion process and the baseline of the autocorrelation, the autocorrelation spectrum of FePc/CoPc were appropriately fitted with few fitting residual errors. Meanwhile, different single-atom catalyst models with multiple catalytic sites were carried out through altering the proportions of the scattered FePc and CoPc molecules, and to simulate the random

distribution and content of different single-atom catalytic sites. In this way, we can further verify the accuracy and range of the SAC-FCS method.

As shown in the representative autocorrelation spectra and the fitting curves of FePc/CoPc models with different proportions (Supplementary Fig. 4), they have the similar trends and distribution of the lag time, and fine fitting with the autocorrelation equation, suggesting that the fitting equation of autocorrelation can well describe the experimental data of fluorescence autocorrelation spectra. Thus, we can obtain the corresponding kinetics parameters, such as the proportion of active sites (r_1) with fast catalytic reaction process. Through 500 times analysis and fitting of the fluorescent trajectories from different illuminated regions, we get the statistic proportional distribution of the active sites of each FePc/CoPc model (Supplementary Fig. 5), which have the narrow distribution and are in close to the actual contents of the FePc active sites. The above FePc/CoPc models demonstrated the universality of the SAC-FCS method for the single-atom catalyst system with double catalytic sites.

Then we further assessed the applicability of the SAC-FCS method for a more complicated ternary FePc/CoPc/NiPc system in the same way to simulate the real SACs with multiple catalytic sites. The contents of FePc in these ternary systems were successively changing from 10% to 90%. Supplementary Fig. 6 displayed one of the autocorrelation spectra and the corresponding fittings of each proportion, which can be fitted well with the SAC-FCS model with small errors. Meanwhile, the measured FePc ratios had narrow distributions and were close to the actual values in these FePc/CoPc/NiPc models (Supplementary Fig. 7). These indicate the high accuracy of the SAC-FCS method for recognizing the actively atomic sites at a wide range. Based on the above standard single-atom catalyst models with binary and ternary catalytic sites, we successfully identified and quantified the active sites of the FePc molecules through the SAC-FCS method, which showed the great potentials and significance of the SAC-FCS method for revealing the contents and kinetic behaviors of the inherent active sites in SACs with multiple catalytic sites.

3.2. Structural Characterization of FeN_x/C SACs

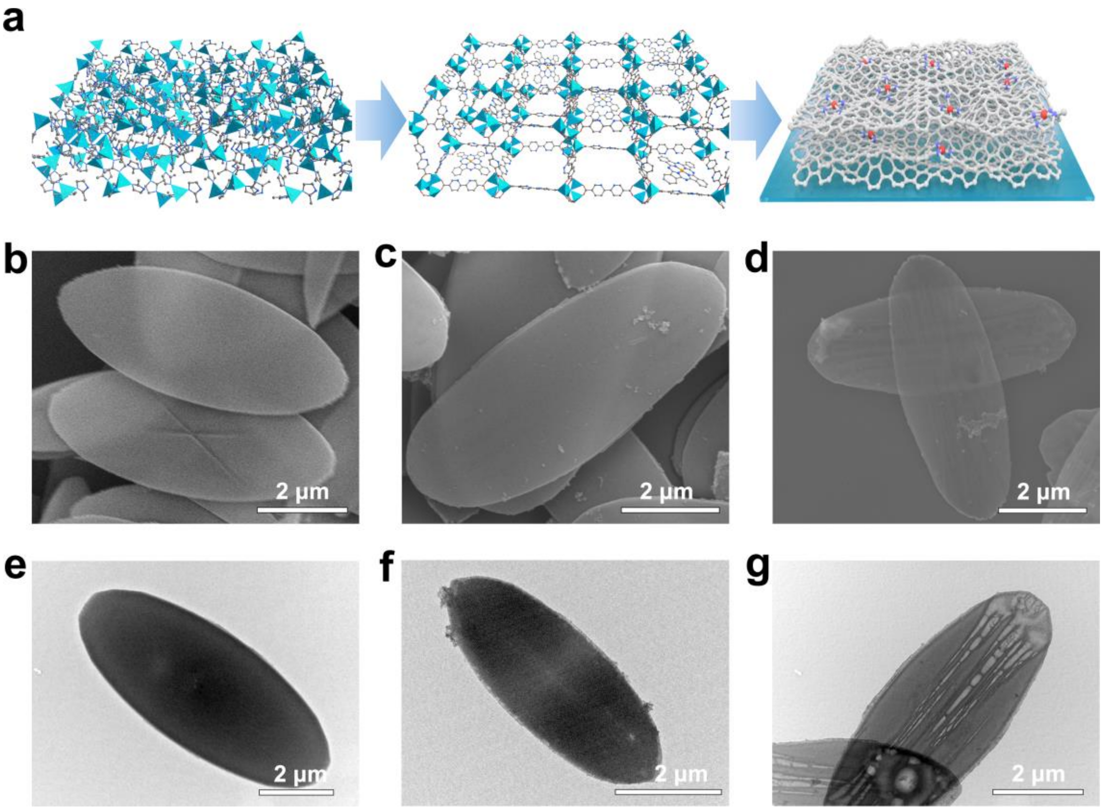

Supplementary Fig. 8 | Synthetic scheme and morphology characterization of the MOFs precursors and FeN_x/C SACs. (a) Schematic formation process of FeN_x/C single-atom catalyst models through the strategies of ligand exchange, encapsulation and pyrolysis of the MOFs precursors. The blue tetrahedron represents the Zn-N(O) clusters in MOFs. The red and blue balls respectively represent the Fe and N atoms in SACs. (b-d) Representative SEM images and (e-g) TEM images of the individual nanosheets of the ZIF-L, Zn-MOF and FeN_x/C SACs, respectively.

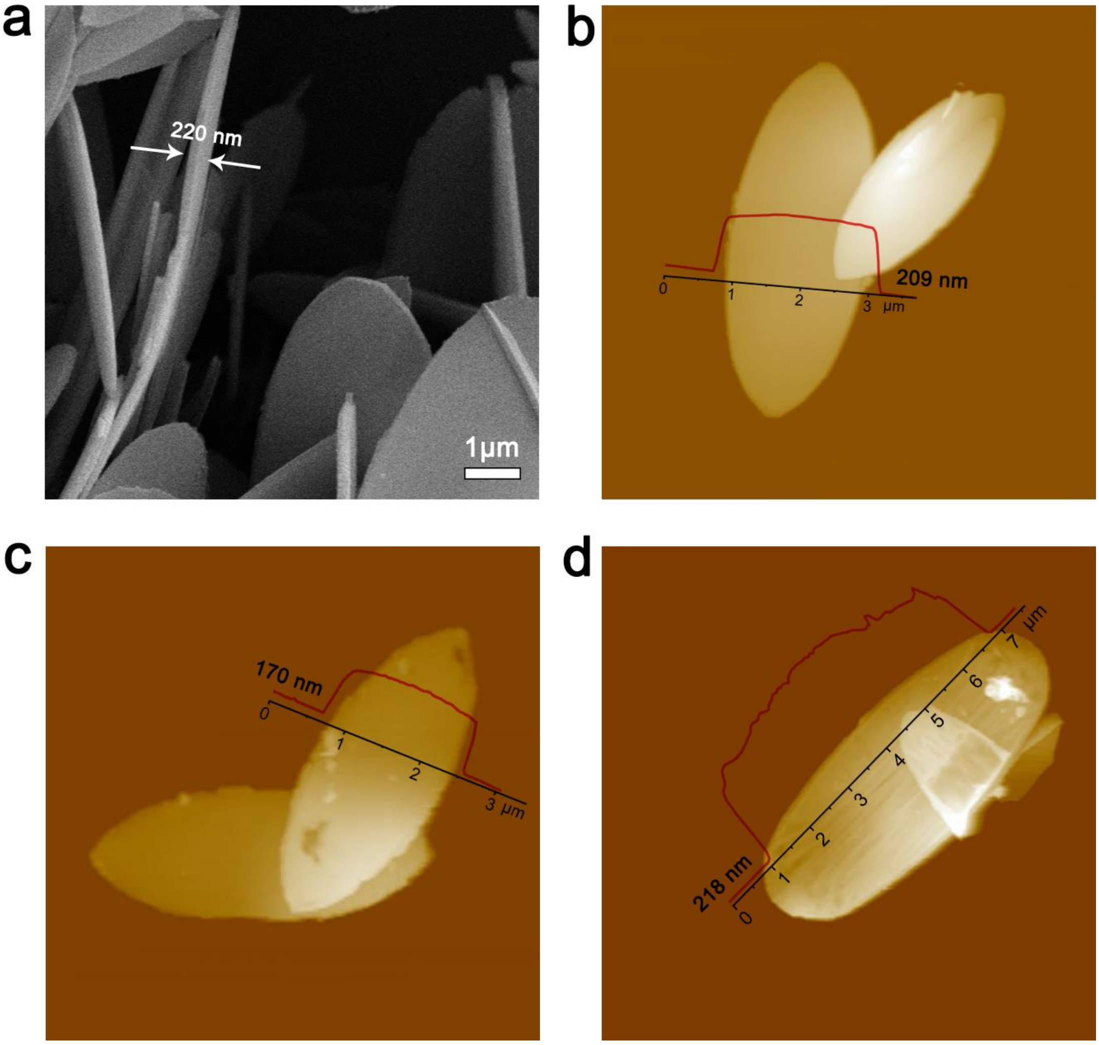

Supplementary Fig. 9 | Thickness of the nanosheets. (a) SEM image of ZIF-L and typical AFM images of (b) ZIF-L, (c) Zn-MOF and (d) FeN_x/C SACs.

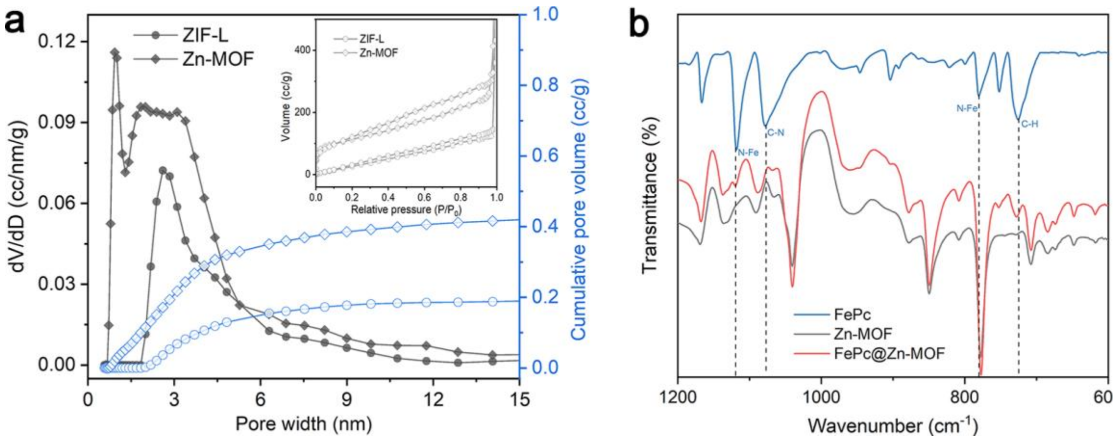

Supplementary Fig. 10 | Pore structure and composition characterization of ZIF-L and Zn-MOF precursors. (a) Pore size distribution of FePc@Zn-MOF, and the inset is the corresponding N_2 adsorption/desorption isotherms. (b) FTIR spectra of FePc, Zn-MOF, and FePc@Zn-MOF.

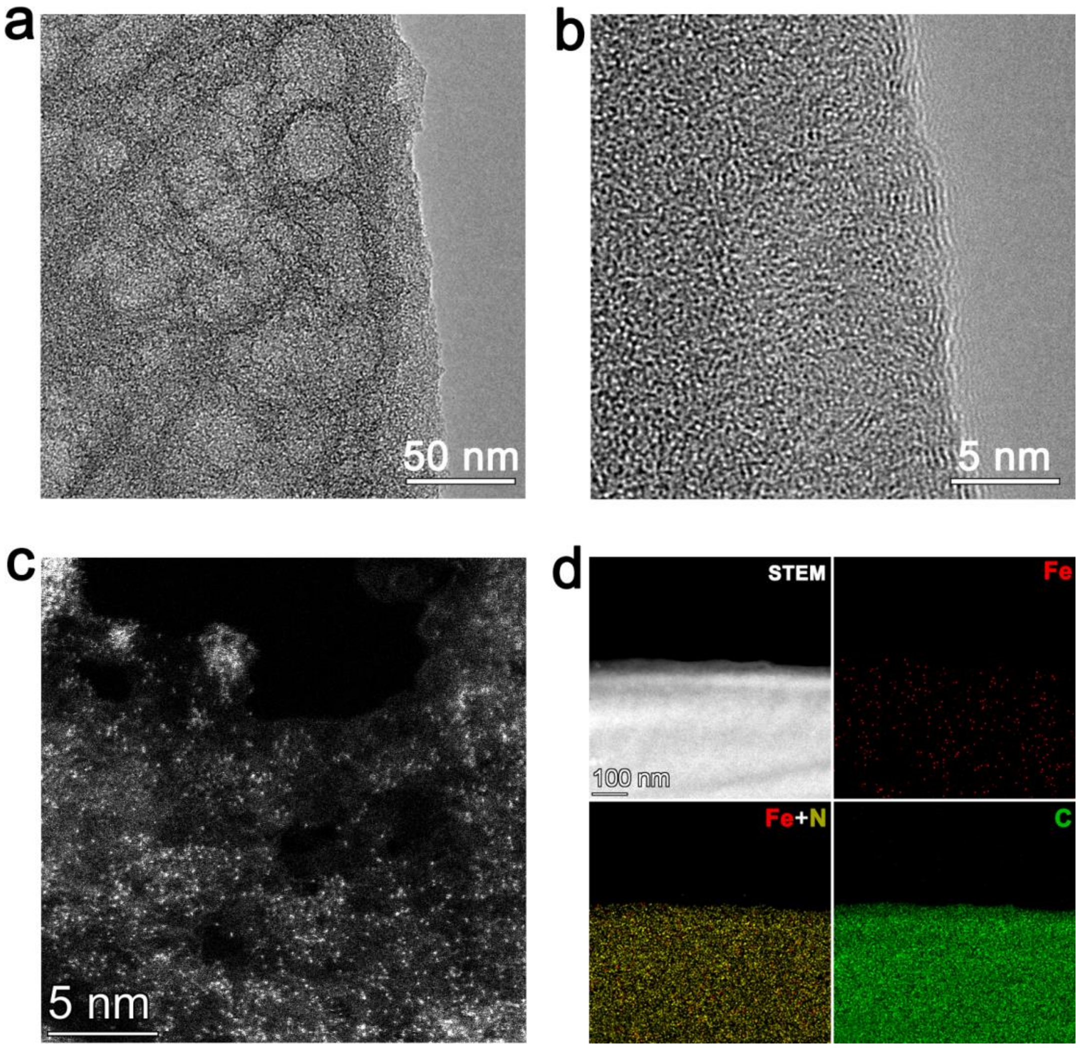

Supplementary Fig. 11 | Atoms and elements distribution of FeN_x/C SACs. (a) TEM and (b) HRTEM images of FeN_x/C-3 SACs. (c) HAADF-STEM image of the Fe single atoms and (d) the corresponding EDS mappings of the distribution of Fe, N and C elements.

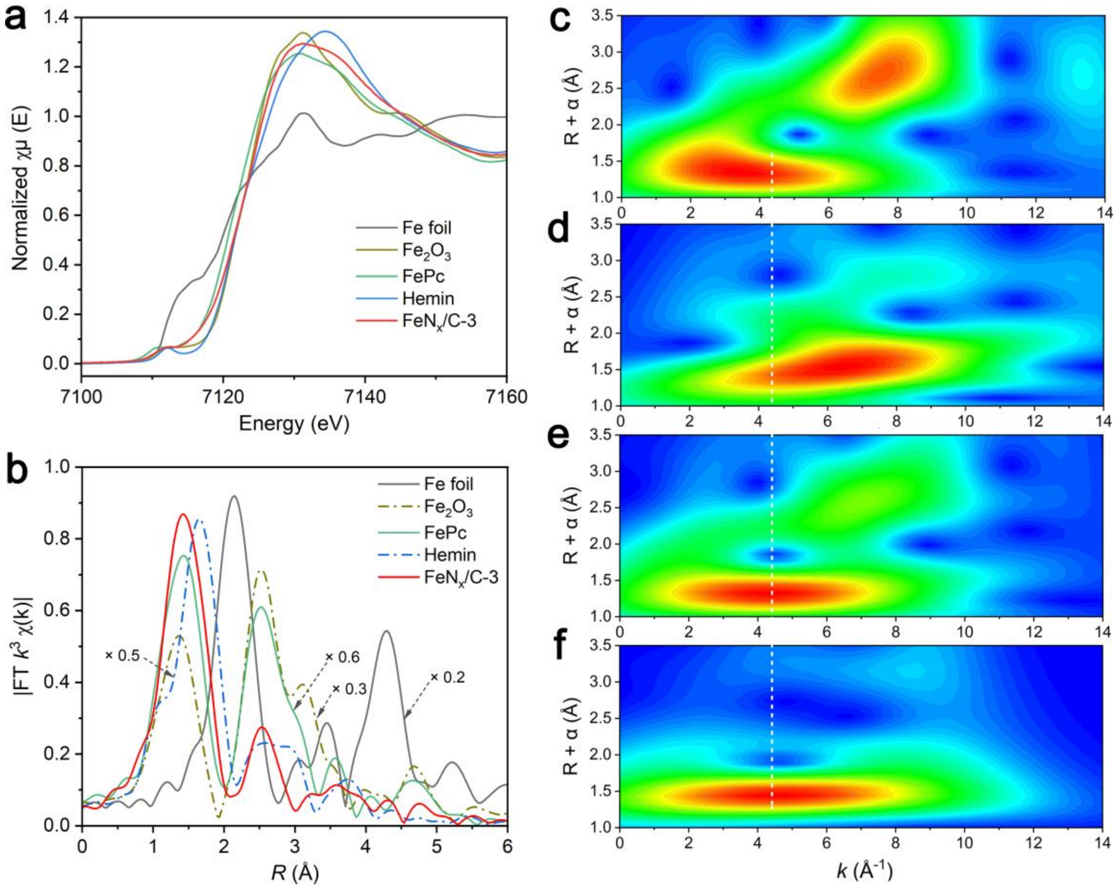

Supplementary Fig. 12 | Atomic structure characterization of FeN_x/C SACs. (a) Normalized XANES spectra at Fe K-edge of the Fe foil, Fe_2O_3, FePc, Hemin and FeN_x/C-3 SACs and (b) the corresponding Fourier transformations for the k^3-weighted Fe K-edge EXAFS in R-space. Wavelet transforms for the k^3-weighted Fe K-edge EXAFS of (c) Fe_2O_3, (d) Hemin, (e) FePc and (f) FeN_x/C-3 SACs.

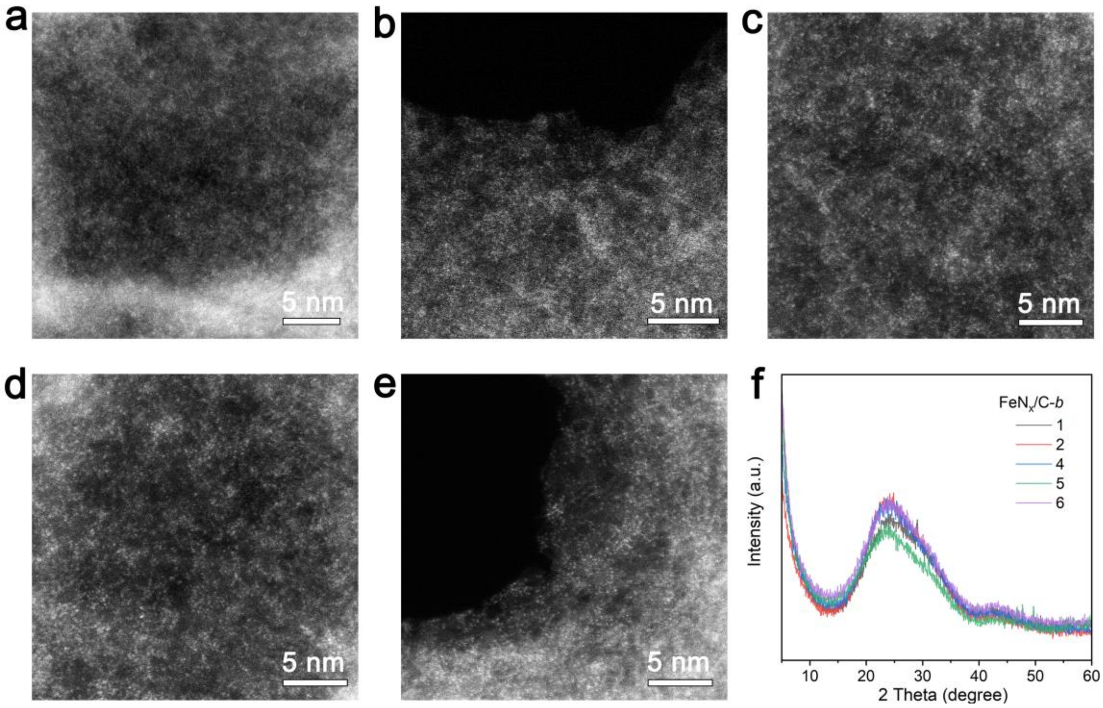

Supplementary Fig. 13 | Fe single-atom characterization of the other FeN_x/C SACs models. (a-e) HAADF-STEM images of the FeN_x/C-*b* SCAs, respectively, *b*=1, 2, 4, 5 and 6, showing the dominant Fe single atoms, and (f) the corresponding XRD patterns of FeN_x/C-*b* SCAs.

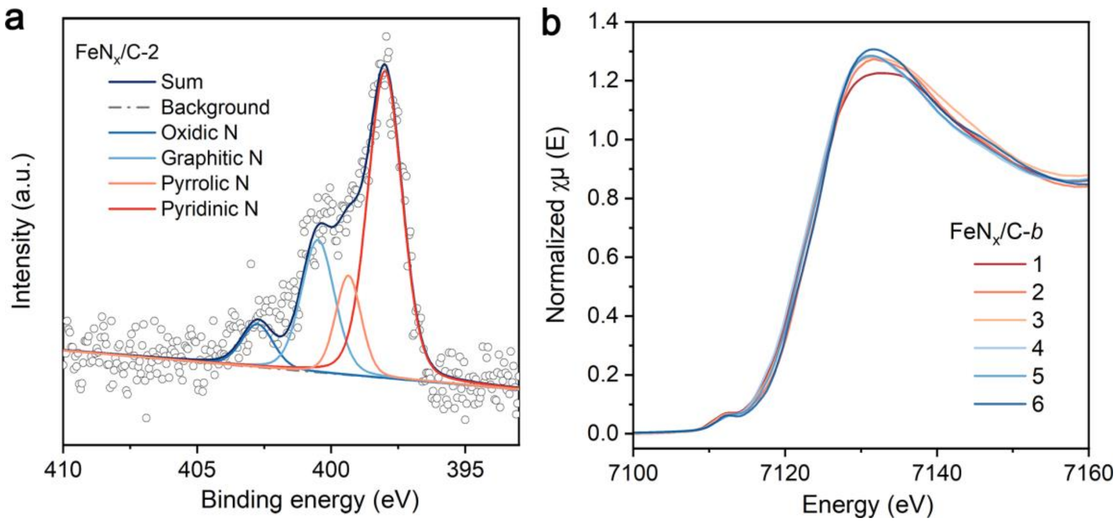

Supplementary Fig. 14 | XPS and XANES spectra of FeN_x/C SACs. (a) High-resolution XPS spectrum of N *1s* of FeN_x/C-2. (b) Normalized XANES spectra at Fe K-edge of the FeN_x/C-*b* SACs.

Supplementary Discussion 3.2

We synthesized a series of the FeN_x/C SACs with the controllable loading of the Fe single atoms through the MOFs pyrolysis strategy. As shown in Supplementary Fig. 8, the two-dimensional ZIF-8 nanosheets with large area and uniform thickness were first chosen as the MOFs templates. And then the Zn-MOFs nanosheets were formed by ligand exchange and templated growth, the reconstruction of framework structure enabled the in-situ encapsulation of FePc to form the SACs precursors (FePc@Zn-MOF). After the pyrolysis and reconstruction strategies, the precursors transformed into the FeN_x/C SACs with randomly distributed atomic and coordinate structures. After the ligand exchange and the pyrolysis, the SEM images indicated that the FeN_x/C SACs still maintained the original flake-like microstructure with the average length of 3 μm. The large-scale two-dimensional plane and the carbon matrices confined FeN_x isolated sites of FeN_x/C SACs guaranteed the randomness and consistency between the different observation areas at the single-molecule fluorescence and single-atom level.

The surface and thickness of the precursor and catalysts were further characterized by atomic force microscopy (AFM). As shown in Supplementary Fig. 9, the MOFs and FeN_x/C SACs had the uniform thickness spread over the two-dimensional plane, which were about 300 nm and within the detection depth of the single-molecule fluorescence microscopy, thus made the effective capture of the fluorescence signal across the vertical volume.

As shown in Supplementary Fig. 10, the Brunauer-Emmet-Teller (BET) nitrogen adsorption/desorption technique demonstrated the expansion of the specific surface area and pore diameter, which enabled the controllable immobilization of the homo-dispersed FePc sites. The characteristic peaks of FePc@Zn-MOF in Fourier transform infrared (FT-IR) spectrum also indicated the successful encapsulation of FePc.

We further characterized the atomic structures of the FeN_x/C SACs through various X-ray spectra. As shown in Supplementary Fig. 11, the HRTEM images exhibited no observable metal particles, excluding the form of Fe nanoparticles in carbon framework. The aberration-corrected STEM images of FeN_x/C-3 confirmed the existence of atomically dispersed Fe atoms over the carbon nanosheets, and the observation of multiple regions indicates that only individual Fe metal atoms (Supplementary Fig. 11c). The corresponding EDS mapping images showed that the Fe and N atoms are homogeneously distributed throughout the whole domain, further indicated the single-atom sites of the catalyst (Supplementary Fig. 11d).

Then we conducted X-ray absorption fine structure (XAFS) for the coordinately

atomic structural analysis of the FeN_x/C-3 SACs. As shown in Supplementary Fig. 12a, the Fe K-edge X-ray absorption near-edge structure (XANES) profile of FeN_x/C-3, with Fe foil, Fe_2O_3, Hemin and FePc as references, was located between the Hemin and FePc, revealing a higher oxidation valence state of the atomically dispersed Fe species at +2~+3, which meant that the more electron transfers from Fe atoms to the coordination N atoms compared to the square planar FeN_4 structure. The Fourier-transformed (Supplementary Fig. 12b) and wavelet-transformed (Supplementary Fig. 12c-f) k^3-weighted extended XAFS (FT-EXAFS) spectrum of FeN_x/C-3 at Fe K-edge presented a main peak at 1.50 Å, which was in accord with Fe-N scattering path of the FePcCl and FePc, and no Fe-O bond or Fe-Fe bond was detected, indicating the formation of the atomically dispersed Fe-N_x sites in FeN_x/C-3.

Meanwhile, the atomic Fe loading of the SACs could be altered by the added amount of the FePc precursor during the synthetic process. Then we prepared a series of FeN_x/C-*b* SACs with different amounts of Fe atomic sites for the systematic research of the interrelation between the single-atom loading and the reaction kinetics through the SAC-FCS method.

In accordance with the FeN_x/C-3, the other FeN_x/C-*b* SACs also presented with the atomically dispersed Fe atoms on the substrates (Supplementary Fig. 13). Obviously, with the increase of the Fe loading, the densities of the single-atom Fe in STEM images gradually increase without any metal clusters or nanoparticles, in accord with the no observable characteristic peaks in XRD patterns, indicating that the confinement of the MOFs precursor was conducive to atomic dispersion.

The XPS N 1s spectra of these FeN_x/C-*b* SACs were principally deconvoluted into pyridinic N and pyrrole N (Supplementary Fig. 14), which derived from dipyridine linkers and phthalocyanine molecules, contributed to the coordination with Fe atoms and the form of multiple FeN_x spatial structures. Correspondingly, the Fe K-edge XANES profiles of these FeN_x/C-*b* SACs share the similar oxidation states, indicating the uniformity of the atomically dispersed FeN_x sites even with the variation of the metal loading.

3.3 SAC-FCS analysis for FeN_x/C SACs

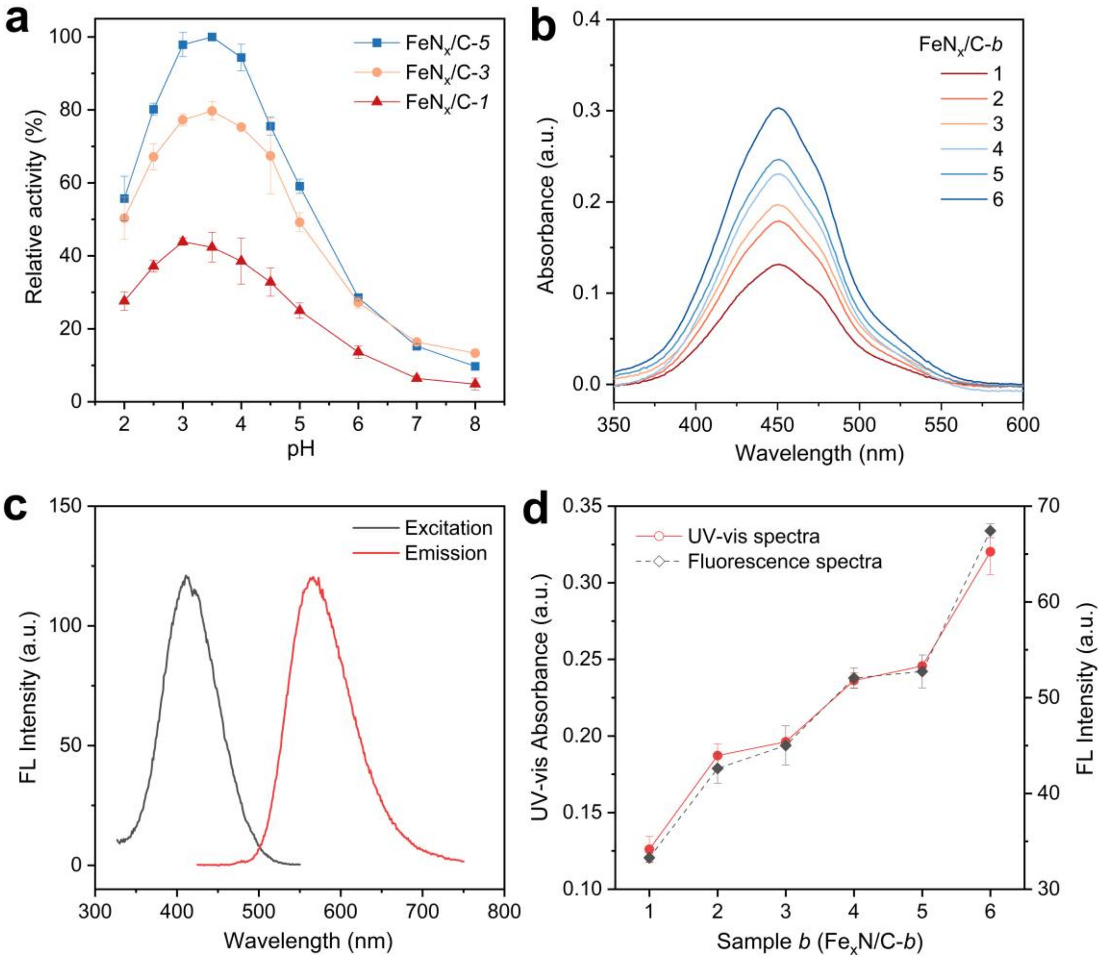

Supplementary Fig. 15 | Oxidase-like activity of FeN_x/C SACs models. (a) pH-dependent oxidase-like activity of FeN_x/C-*b* SACs in air-saturated buffer. (b) UV-vis absorbance changes at 450 nm by using FeN_x/C-*b* SACs as oxidase mimics. (c) Fluorescence excitation and emission spectra of the OPD. (d) Catalytic activity of FeN_x/C-*b* (*b* = 1,2,3,4,5,6) respectively indicated by the UV-vis absorbance and fluorescence intensity of OPD.

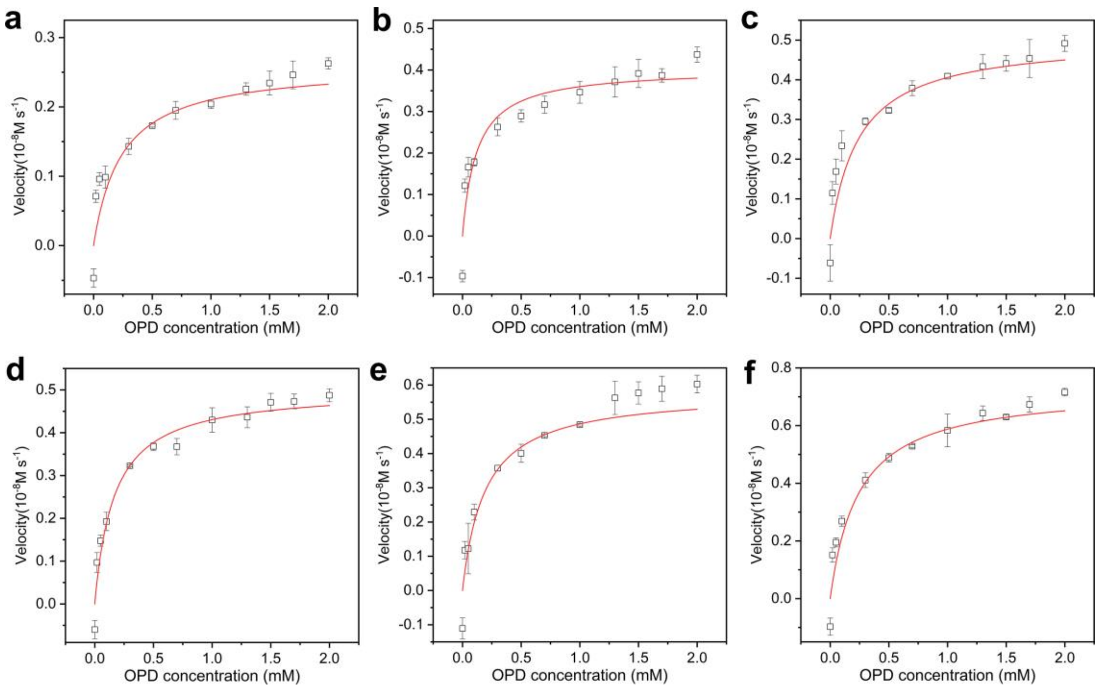

Supplementary Fig. 16 | Oxidase-like catalytic kinetics of FeN*x*/C SACs. (a-f) Michaelis-Menten curves of OPD oxidation catalyzed by a series of FeN_x/C-*b* SACs, respectively, *b* = 1,2,3,4,5,6.

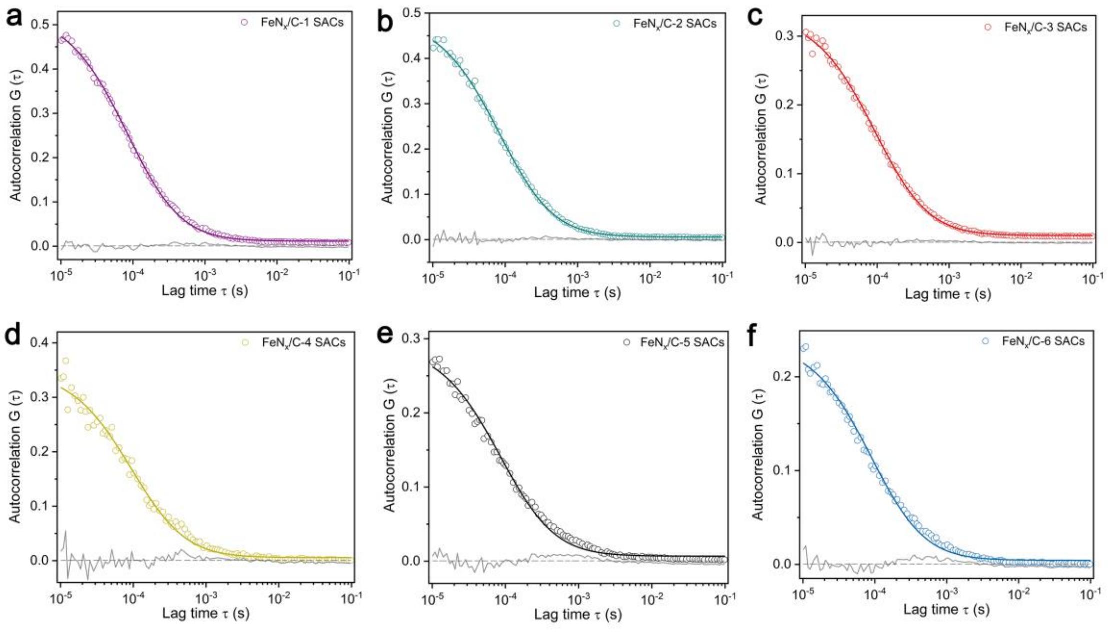

Supplementary Fig. 17 | Autocorrelation data and fitting curves of the FeN_x/C SACs models. (a-f) Typical fluorescence signals and autocorrelation of a series of FeN_x/C-*b* SACs, respectively, *b* = 1,2,3,4,5,6. The circles represent the autocorrelation data from experiments and solid lines are fittings of *Equation 20* to the data, and gray lines are the corresponding errors of the fittings.

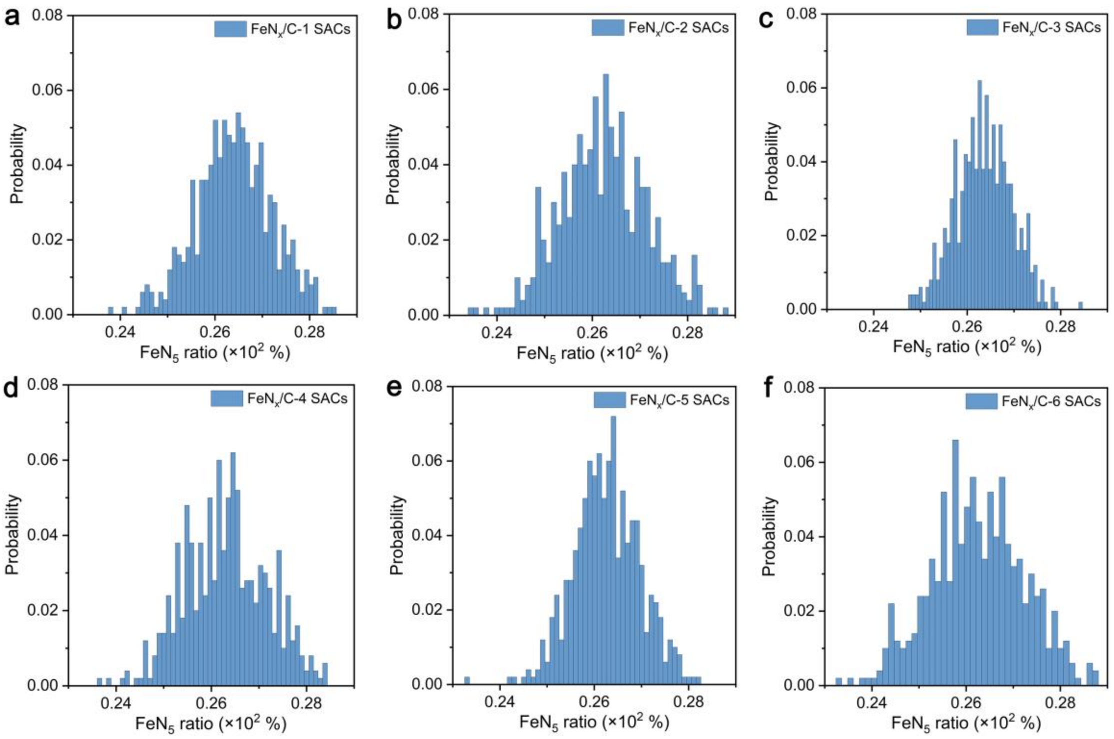

Supplementary Fig. 18 | SAC-FCS fitting ratio of FeN_5 catalytic sites in FeN_x/C SACs model. (a-f) Intensity histogram of the fitting ratio of the FeN_5 catalytic sites in different FeN_x/C-*b* SACs catalytic models, where *b* represents the different loading of the Fe single atoms in each model.

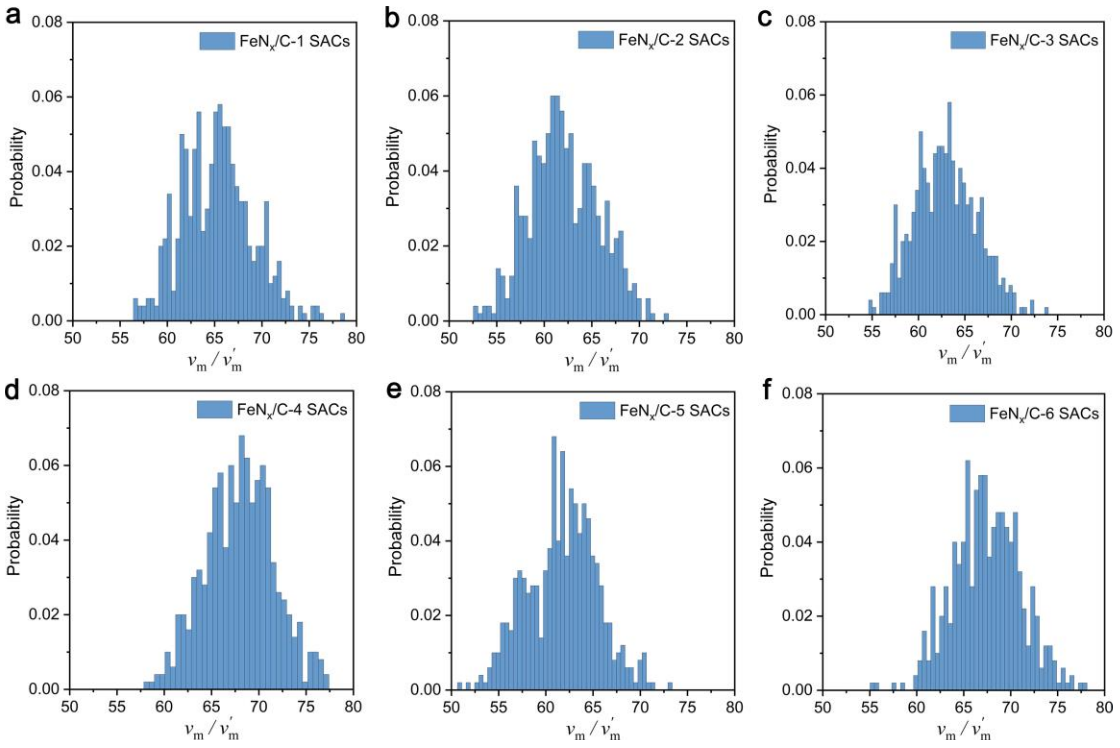

Supplementary Fig. 19 | SAC-FCS fitting kinetics of the single-atom catalytic rate in FeN$_x$/C SACs model. (a-f) Intensity histogram of the fitting kinetics of different single-atom catalytic sites in different FeN$_x$/C-*b* SACs catalytic models. The v_m/v_m' represents the ratio of the maximum reaction rates between FeN$_5$ and FeN$_{4/6}$ single-atom sites.

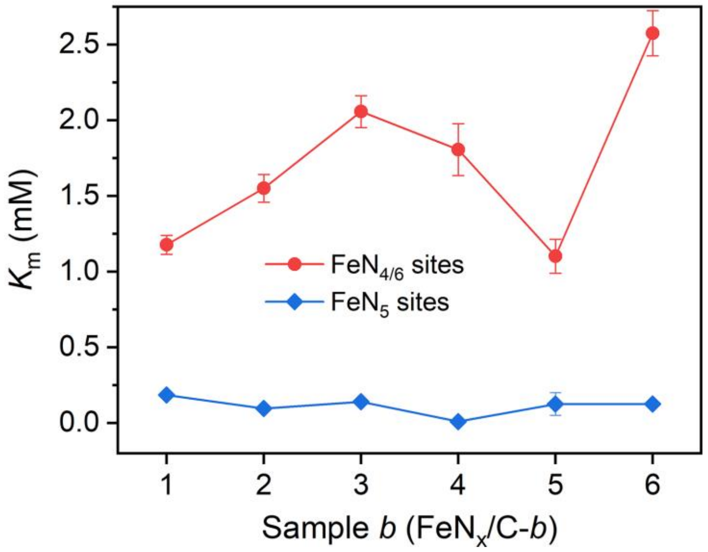

Supplementary Fig. 20 | Theoretical investigation of the intrinsic active sites in FeN_x/C SACs model. SAC-FCS fitting K_m of FeN_5 and $FeN_{4/6}$ single-atom sites in FeN_x/C SACs.

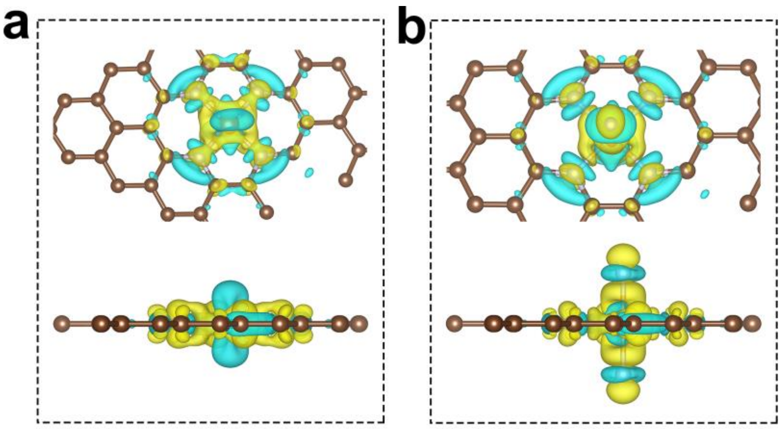

Supplementary Fig. 21 | Theoretical investigation of the intrinsic active sites in FeN_x/C SACs model. DFT calculation of charge density difference plots (top and front view) for (a) FeN_4 and (b) FeN_6 single-atom sites in FeN_x/C SACs. (c) SAC-FCS fitting K_m of FeN_5 and $FeN_{4/6}$ single-atom sites in FeN_x/C SACs.

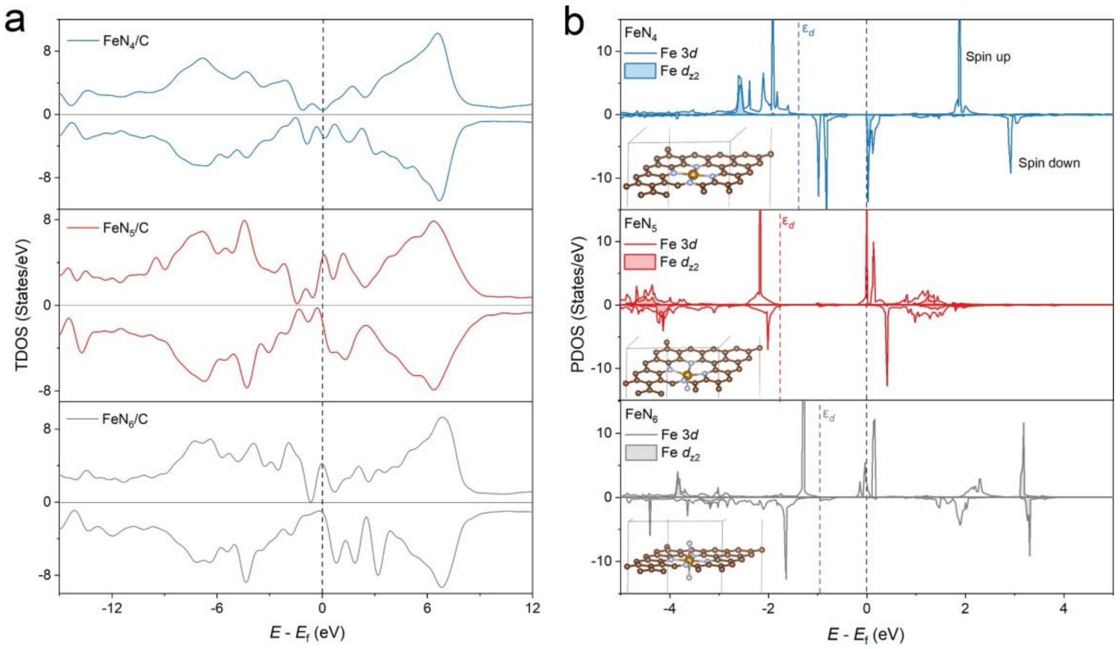

Supplementary Fig. 22 | DOS of FeN_x/C SACs. (a) Total density of states (TDOS) of FeN_4/C, FeN_5/C and FeN_6/C sites. The vertical dashed line at the zero is at the Fermi energy level. (b) Partial density of states (PDOS) of Fe 3*d*- and d_{z^2} orbitals of the three FeN_x/C. The ε_d represents the location of Fe *d*-band center. The insert ball-and-stick models in (b) represent FeN_x/C structures, where brown, blue and yellow balls represent the C, N and Fe atoms, respectively.

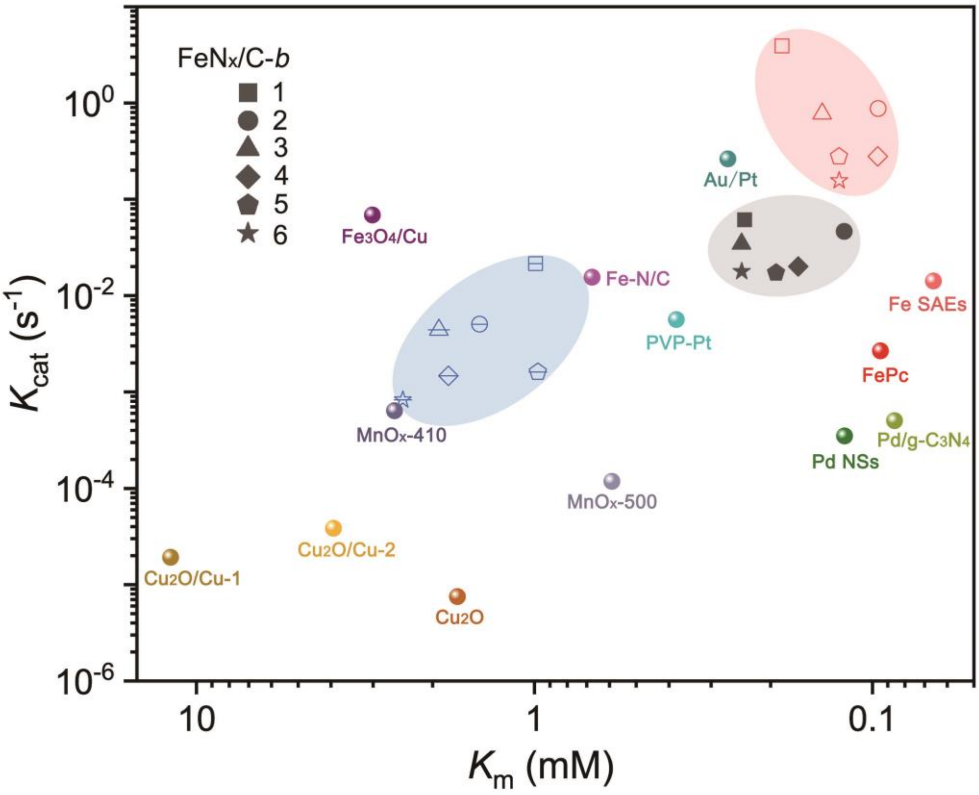

Supplementary Fig. 23 | Single-atom kinetics of the SACs. The comparison of the atomic oxidase-like kinetics of the FeN_x/C-*b* SACs and the other metal-based catalysts. The black, red and blue zones respectively represent the apparent activity distribution of the FeN_x single-atom sites and the atomic kinetic distribution of the FeN_5 and $FeN_{4/6}$ sites in FeN_x/C-*b* SACs. K_m is the Michaelis constant. K_{cat} is the catalytic constant, which is calculated by SAC-FCS fitting or normalized by the molar concentration of the active atom according to the references. FePc: Iron phthalocyanine. Fe-N/C: Fe-N/C SACs[6]. Fe SAEs: Fe single atom enzymes[7]. Fe_3O_4/Cu: Fe_3O_4@Cu/guanosine 5′-monophosphate[8]. Cu_2O: Cu_2O in light irradiation, Cu_2O/Cu-1: Cu_2O-carbon dots-Cu in the dark condition, Cu_2O/Cu-2: Cu_2O-carbon dots-Cu in light irradiation[9]. MnO_x-410: MnO_x microspheres with 410 °C calcination, MnO_x-500: MnO_x microspheres with 500 °C calcination[10]. Au/Pt: Au@Pt core-shell nanoparticles[11]. PVP-Pt: polyvinylpyrrolidone-stabilized Pt nanoclusters[12]. Pd/g-C_3N_4: graphitic carbon nitride nanosheets/Pd nanosheets, Pd NSs: Pd nanosheets[13].

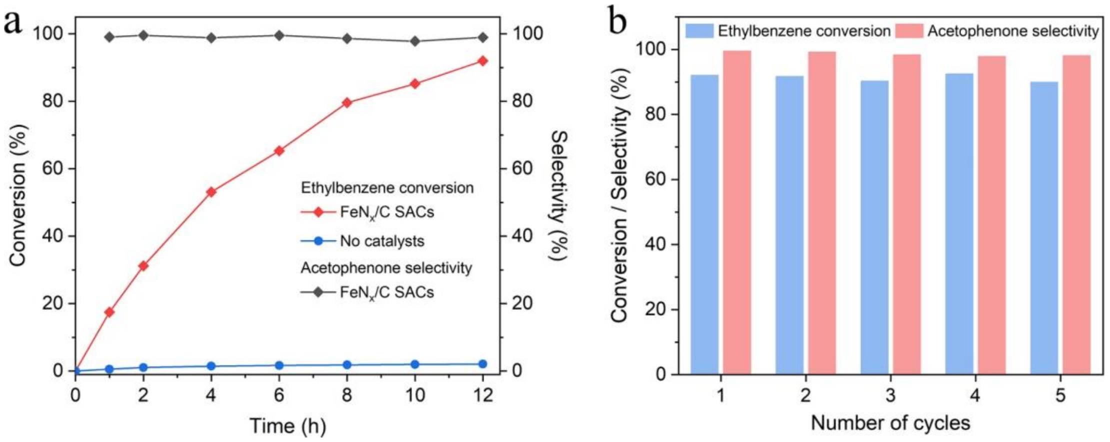

Supplementary Fig. 24 | Evolution of catalytic performance and stability of FeN_x/C SACs. (a) Time-dependent conversion/selectivity profile for ethylbenzene oxidation. (b) Recycling of FeN_x/C SACs.

Supplementary Table 1 | Mössbauer parameters. The ^{57}Fe Mössbauer parameters and proportion of different Fe species in FeN_x/C-*b* SACs.

Sample	Fe species[a]	δ_{iso} (mm s^{-1})[b]	ΔE_Q (mm s^{-1})[c]	Area (%)[d]
	D1	0.31	0.91	48.41
FeN_x/C-1	D2	0.49	2.21	29.45
	D3	1.15	2.55	22.14
	D1	0.32	1.16	45.05
FeN_x/C-2	D2	0.48	2.37	31.60
	D3	1.14	2.50	23.35
	D1	0.29	1.18	43.35
FeN_x/C-3	D2	0.45	2.46	31.90
	D3	1.15	2.45	24.75
	D1	0.28	1.28	44.04
FeN_x/C-4	D2	0.51	2.42	31.46
	D3	1.16	2.58	24.50
	D1	0.29	1.30	39.06
FeN_x/C-5	D2	0.44	2.31	35.90
	D3	1.19	2.40	25.04
	D1	0.31	1.31	43.48
FeN_x/C-6	D2	0.47	2.34	32.03
	D3	1.14	2.57	24.49

[a] The Mössbauer parameters of doublets (D1, D2, D3) respectively correspond to the FeN_4, FeN_6 and FeN_5 species. [b] Mössbauer isomer shift (*IS*, δ_{iso}) is a relative measure describing a shift in the resonance energy of a nucleus, relative to α-iron. [c] Quadrupole splitting (*QS*, ΔE_Q) reflects the interaction between the nuclear energy levels and the surrounding electric field gradient. [d] The relative absorption area of the different iron species in FeN_x/C-*b* SACs samples.

Supplementary Table 2 | Atomic kinetics. The comparison of the ensemble and relatively atomic oxidase-like kinetics of different FeN_x/C-*b* SACs from experimental colorimetric assays and SAC-FCS autocorrelation fitting.

Sample	Fe content (wt%)	Activity assays		Autocorrelation fitting	
		v_m (10^{-8} M s^{-1})[a]	K_m (mM)[b]	v_m/v_m'	K_m/K_m'
FeN_x/C-1	0.0237	0.261	0.239	65.248	0.187
FeN_x/C-2	0.0458	0.404	0.121	62.073	0.066
FeN_x/C-3	0.0815	0.505	0.244	62.917	0.073
FeN_x/C-4	0.1395	0.502	0.166	68.032	0.054
FeN_x/C-5	0.1877	0.581	0.193	61.739	0.128
FeN_x/C-6	0.2280	0.731	0.243	67.438	0.051

[a] v_m is the maximal reaction velocity. [b] K_m is the Michaelis constant.

Supplementary Table 3 | Oxidation of ethylbenzene to acetophenone catalyzed by different catalysts.

Catalyst	Metal content (wt%)	[E] (mM)	Conversion (%)	Selectivity (%)	TOF (h^{-1})	Ref.
FeN_x/C SACs	0.0815	0.146	92	99	263.4	This work
Co SACs	23.58	8.4	46	97	19.6	[14]
Co nanoparticle	1.14	4.2	81	98	10.66	[15]
$CoBr_2$	26.9	32.9	42	30	11.4	[16]
Co_3O_4	73.4	12.4	42	90	87.8	[17]
SA-Fe–TCN	1.3	1.55	39	95	7.0	[18]
Fe-PHI	0.1	0.22	99.6	98.4	65.8	[19]
Fe@CNTs	17.3	8.6	36.8	60.2	48.7	[20]
$Mn(OH)_x/\gamma\text{-}Al_2O_3$	1.0	15.0	74	86	41.1	[21]
$\delta\text{-}MnO_2$	63.2	46.0	72.7	92.1	31.0	[22]
Mn nanocatalyst	1.94	7.0	53	74	3.8	[23]

Supplementary Discussion 3.3

As shown in Supplementary Fig. 15, the oxidase-like activity of FeN_x/C-*b* SACs exhibited high pH dependence. With optimizing the pH and substrate concentration, the oxidase-like catalytic reaction of FeN_x/C-*b* SACs were conducted in air-saturated sodium acetate–acetic acid buffer with optimized pH. Meanwhile, due to the UV-vis absorbance and fluorescence emission characteristic peaks of the oxidative product (DAP) at 450 nm and 565 nm, respectively, the oxidase-like activity can be parallelly determined by colorimetric assays of the UV-vis absorbance and fluorescence spectra. As shown in Supplementary Fig. 15d, the consistent trends of the activity changes of FeN_x/C-*b* SACs in the two spectra have demonstrated the effectiveness and reliability of the oxidase-like catalytic model both in the bulk and single-molecule fluorescence modes. The similar Michaelis-Menten curves of FeN_x/C-*b* SACs also indicated their inherently analogous kinetics and catalytic conditions (Supplementary Fig. 16)

As the established SAC-FCS method had been successfully applied to the single-site catalysis of various FePc/CoPc/NiPc models, we further adopted it for the kinetic analysis of these FeN_x/C SACs in the same way. As shown in Supplementary Fig. 17, we obtained the high-quality and well-organized single molecule fluorescent trajectory and autocorrelation curve of the FeN_x/C-*b* SACs, which were attributed to the large-area carbon sheets, uniformly dispersed Fe single atoms and the kinetics differences between different FeN_x catalytic sites. Similarly, we conducted the SAC-FCS analysis and autocorrelation calculations for each group of FeN_x/C-*b* SACs. The experimental autocorrelation data gathered from multiple periods and fluorescence illuminated regions of different FeN_x/C SACs was fitted well with the autocorrelation equation, indicating the great potentiality and universality of this method for uncovering the intrinsic active sites and reaction kinetics of SACs with multiple catalytic sites.

According to the autocorrelation deduction of the intrinsic FeN_5 active site, most of the proportions (r_1) were distributed over a narrow range, which was about 26.5 ± 1.2% (Supplementary Fig. 18). These indicated that the variation of the FeN_5 proportion in different FeN_x/C SACs has no obvious correlation with the atomic Fe loading, so that the apparent activity of the SACs could be promoted by increasing the loading of the metal single-atoms in a certain range, namely the density of the actual active sites. Meanwhile, the above results and Mössbauer spectra also demonstrated the uniformity of the single-atom distribution in each SACs, as well as the effectivity of the SAC-FCS method.

Furthermore, the catalytic kinetic parameters of the FeN_5 active sites were derived

from the autocorrelation *Equation 21* ($c_1 = Kr_1V_m/K_m$), which provided significant and reliable parametric models for the catalytic activity, atomic structure and mechanism research of the single-atom active sites.

In comparison with the different FeN_x/C SACs, the ratios of relative activity between the active sites and inactive sites were only slightly different (Supplementary Fig. 19). The average specific value of the v_m/v_m' was about 65, which indicated that the compositions of the catalytic sites in FeN_x/C SACs were similar, and the unit activity of FeN_5 site outclassed the other single-atom sites. The SAC-FCS method was also proved to be effective and accurate for the recognition of the intrinsic active sites in SACs.

In addition to the calculations of charge density differences (Supplementary Fig. 20), we further calculated the total density of states (TDOS) of FeN_4/C, FeN_5/C and FeN_6/C sites, and the partial density of states (PDOS) of the central Fe atoms. The plotted Fermi level exhibited a negative shift from -1.77 eV to -2.41 eV with the increase of the coordination number (Figure S21a), indicating the strong interactions between Fe and N atoms. Then we investigated the deconvoluted Fe 3*d* orbitals, which are close relevant to the absorption of oxygenated (*OOH*, *O and *OH) intermediates and catalytic activity, with the descriptor of *d*-band center (ε_d). The ε_d shift of active sites is highly associated with different adsorption strengths of intermediates. As shown in Figure S21b, a more positive ε_d (-1.3 eV) of FeN_4 site will give rise to much stronger adsorption of the intermediates than that of the FeN_5/C (ε_d = -1.9 eV), as a result, hydrogenation and desorption of intermediates are retarded. This result is consistent with our previous calculation, for instance, the stronger adsorption of *O on FeN_4/C site resulted in the inert reactivity to *OH[24]. Although the FeN_6/C exhibited a more positive ε_d (-0.9 eV), the steric hindrance from N atoms and the saturated coordination of Fe sites blocked the O_2 adsorption and quenched the reduction at the beginning of reaction. Overall, the configuration and N coordination of FeN_5 sites synergistically optimized the electron and adsorption of Fe 3*d* orbitals, as well as the oxidase-like activity.

We estimated the kinetic parameters of the single-atom sites from the relative ratios of the V_m and K_m (Supplementary Fig. 22). According to the oxidase-like activity and kinetics of FeN_x/C SACs determined by colorimetric assays and SAC-FCS method, we can quantitate the unit activity of the FeN_5 active site from the mixed FeN_x/C SACs (Supplementary Table 2), and make an equipotent comparison of catalytic property between SACs and other enzyme-like catalysts at single-atom level (Supplementary Fig. 23). To evaluate the practical catalytic performance of the catalysts, we selected ethylbenzene as the reactant and systematically investigated the

performance of the FeN_x/C SACs in catalyzing the selective oxidation of ethylbenzene by oxygen. Firstly, a control experiment was performed without catalysts, resulting in only negligible amounts of products. Conversely, under identical conditions, the catalysts of FeN_x/C SACs exhibited extraordinary catalytic performance and selectivity (Supplementary Fig. 24a). As evidenced by the conversion-time curve, the conversion of ethylbenzene exhibited gradually increases over time, while preserving a remarkably high selectivity (>99%) towards acetophenone product (Supplementary Fig. 24a).

When normalizing the catalytic rate to the concentration of FeN_x single-atom sites, the turnover frequency (TOF) of ethylbenzene oxidation on the as-prepared FeN_x/C SACs was much higher than most of the reported catalysts (Table R1), such as Fe-, Co- and Mn-based single atom catalysts, and metal nanoparticles. When the reaction time was extended to 12 hours, the conversion of ethylbenzene reached 92% with an acetophenone selectivity of 99% (Supplementary Fig. 24a). The stability and recyclability of the FeN_x/C SACs were also investigated. As shown in Supplementary Fig. 24b, even after 5 cycles, the conversion (>89%) and selectivity (>98%) did not decrease significantly.

Supplementary References

1. Nørskov, J. K. *et al.* Origin of the Overpotential for Oxygen Reduction at a Fuel-Cell Cathode. *J. Phys. Chem. B* **108**, 17886-17892, (2004).
2. Kresse, G. & Furthmuller, J. Efficient iterative schemes for ab initio total-energy calculations using a plane-wave basis set. *Phys. Rev., B Condens. Matter* **54**, 11169-11186, (1996).
3. Kresse, G. & Furthmüller, J. Efficiency of ab-initio total energy calculations for metals and semiconductors using a plane-wave basis set. *Comput. Mater. Sci.* **6**, 15-50, (1996).
4. Liu, G. *et al.* Pervaporation performance comparison of hybrid membranes filled with two-dimensional ZIF-L nanosheets and zero-dimensional ZIF-8 nanoparticles. *J. Membr. Sci.* **523**, 185-196, (2017).
5. Jiang, B. *et al.* Standardized assays for determining the catalytic activity and kinetics of peroxidase-like nanozymes. *Nat. Protoc.* **13**, 1506-1520, (2019).
6. Jing, W. *et al.* Fe-N/C single-atom nanozyme-based colorimetric sensor array for discriminating multiple biological antioxidants. *Analyst* **146**, 207-212, (2021).
7. Zhao, C. *et al.* Unraveling the enzyme-like activity of heterogeneous single atom catalyst. *Chem. Commun.* **55**, 2285-2288, (2019).
8. Zhang, S. *et al.* Robust magnetic laccase-mimicking nanozyme for oxidizing o-phenylenediamine and removing phenolic pollutants. *J. Environ. Sci. (China)* **88**, 103-111, (2020).
9. Li, F. *et al.* A Cu2O-CDs-Cu three component catalyst for boosting oxidase-like activity with hot electrons. *Chem. Eng. J.* **382**, (2020).
10. Babacan Tosun, R., Kip, Ç. & Tuncel, A. Polymeric template assisted synthesis of monodisperse-porous manganese oxide microspheres: a new nanozyme with oxidase-like activity allowing biomolecule determination via bimodal sensing. *New J. Chem.* **43**, 18505-18516, (2019).
11. Bonet-Aleta, J., Garcia-Peiro, J. I., Irusta, S. & Hueso, J. L. Gold-Platinum Nanoparticles with Core-Shell Configuration as Efficient Oxidase-like Nanosensors for Glutathione Detection. *Nanomaterials (Basel)* **12**, (2022).
12. Liu, X., Tian, M., Li, C. & Tian, F. Polyvinylpyrrolidone-stabilized Pt nanoclusters as robust oxidase mimics for selective detection of ascorbic acid. *Colloids Surf. A Physicochem. Eng. Asp.* **625**, (2021).
13. Zhang, C. *et al.* Enhanced oxidase-like activity of g-C3N4 nanosheets supported Pd nanosheets for ratiometric fluorescence detection of acetylcholinesterase activity and its inhibitor. *Chin. Chem. Lett.* **33**, 757-761, (2022).
14. Xiong, Y. *et al.* Cobalt single atom site catalysts with ultrahigh metal loading for enhanced aerobic oxidation of ethylbenzene. *Nano Res.* **14**, 2418-2423, (2021).

15. Habibi, D., Faraji, A. R., Arshadi, M., Heydari, S. & Gil, A. Efficient catalytic systems based on cobalt for oxidation of ethylbenzene, cyclohexene and oximes in the presence of N-hydroxyphthalimide. *Appl. Catal. A-Gen.* **466**, 282-292, (2013).
16. Gutmann, B., Elsner, P., Roberge, D. & Kappe, C. O. Homogeneous Liquid-Phase Oxidation of Ethylbenzene to Acetophenone in Continuous Flow Mode. *ACS Catal.* **3**, 2669-2676, (2013).
17. Liu, Y. *et al.* Efficient Selective Oxidation of Aromatic Alkanes by Double Cobalt Active Sites over Oxygen Vacancy-rich Mesoporous Co3O4. *Angew. Chem. Int. Ed.*, e202306261, (2023).
18. Xiao, X. *et al.* A Unique Fe-N(4) Coordination System Enabling Transformation of Oxygen into Superoxide for Photocatalytic C-H Activation with High Efficiency and Selectivity. *Adv. Mater.* **34**, e2200612, (2022).
19. da Silva, M. A. R. *et al.* Sustainable oxidation catalysis supported by light: Fe-poly (heptazine imide) as a heterogeneous single-atom photocatalyst. *Appl. Catal. B-Environ.* **304**, (2022).
20. Luo, J., Yu, H., Wang, H. & Peng, F. Enhancing the catalytic activity of carbon nanotubes by filled iron nanowires for selective oxidation of ethylbenzene. *Catal. Commun.* **51**, 77-81, (2014).
21. Kuwahara, Y., Yoshimura, Y. & Yamashita, H. In situ-created Mn(iii) complexes active for liquid-phase oxidation of alkylaromatics to aromatic ketones with molecular oxygen. *Catal Sci Technol* **6**, 442-448, (2016).
22. Liu, J., Wang, H., Wang, L., Jian, P. & Yan, X. Phase-dependent catalytic performance of MnO2 for solvent-free oxidation of ethybenzene with molecular oxygen. *Appl. Catal. B-Environ.* **305**, (2022).
23. Habibi, D., Faraji, A. R., Arshadi, M., Veisi, H. & Gil, A. Manganese nanocatalyst and N-hydroxyphthalimide as an efficient catalytic system for selective oxidation of ethylbenzene, cyclohexene and oximes under aerobic condition. *J. Mol. Catal. A-Chem.* **382**, 41-54, (2014).
24. Huang, L., Chen, J., Gan, L., Wang, J. & Dong, S. Single-atom nanozymes. *Sci. Adv.* **5**, eaav5490, (2019).
